# Identification of physiological races of *Puccinia striiformis *f. sp.* tritici* and molecular docking of some biological treatments as prospective fungal inhibitor candidates in wheat

**DOI:** 10.1038/s41598-026-50602-2

**Published:** 2026-05-06

**Authors:** Hanaa S. Omar, Atef A. Shahin, Mohamed D. Sehsah, Khadegah M. A. Najeeb, Ola Ibrahim Mabrouk, Mai N. Abo El-Yazied, Menna Tallah S. Nady, Mohamed A. Gad

**Affiliations:** 1 Genetics, Faculty of Agriculture, Cairo University, Giza, 12613 Egypt; 2 Agriculture Research Centre (ARC), Plant Pathology Research Institute (PPRI), Giza, 12613 Egypt; 3 Faculty of Medicine, Modern University for Technology and Information (MTI), Cairo, Egypt

**Keywords:** *Triticum aestivum*, Stripe rust, Molecular docking, Biological control, SNP gene, Biotechnology, Genetics, Microbiology, Molecular biology, Plant sciences

## Abstract

Stripe rust caused by *Puccinia striiformis* f. sp. *tritici* (Pst), represents one of the most significant challenges to the world’s wheat production. This study aimed to investigate five aggressive physiological races of Pst impacting significant wheat cultivars in Egypt’s northern Delta, which are essential for breeding programs. Moreover, the study evaluated the antifungal activity and mechanisms of action of chitosan nanoparticles, *Trichoderma harzianum*, and *Sargassum latifolium* against Pst using molecular docking analysis. During the 2024 growing season, an increase in the virulence of newly identified aggressive Pst races in Egypt was detected. A total of 20 and 23 races were recognized from 89 out of 132 samples in 2023 and 86 out of 115 samples in 2024, respectively. In addition, twenty Egyptian wheat cultivars and fifty-two CIMMYT nursery lines were screened at the Sakha Agricultural Research Station for resistance to five aggressive stripe rust races at both seedling and adult stages. The results revealed that most cultivars were susceptible, with only two (10%) and five (25%) displaying resistance. Among the CIMMYT entries, 17 (33%) and 21 (40%) confirmed resistance in 2023 and 2024, respectively. Single nucleotide polymorphism (SNP) markers were used to identify the five Pst races, and phylogenetic analysis was conducted using representative fungal species from genomic databases. The five aggressive races were deposited in the GenBank database under accession numbers PV983376, PV983377, PV983378, PV983379, and PV983380. Biological treatments using *S. latifolium*, *T. harzianum*, and chitosan nanoparticles significantly decreased stripe rust disease severity. These findings recommend that such treatments can support high-quality wheat flour production and serve as safe, environmentally friendly alternatives to synthetic fungicides. Molecular docking analysis further elucidated the mechanisms of antifungal action, particularly highlighting the effectiveness of S*. latifolium* and *T. harzianum* against Pst races. Overall, this investigation exhibits strong potential for improving wheat cultivars with enhanced yield and resistance to aggressive stripe rust races, contributing to sustainable disease management strategies.

**Supplementary Information:**

The online version contains supplementary material available at 10.1038/s41598-026-50602-2.

## Introduction

Wheat is vital for global food security; yet, its production is threatened by several biotic factors in Egypt and worldwide. Among the most significant diseases is yellow (stripe) rust, caused by *Puccinia striiformis*, and leaf rust, caused by *Puccinia triticina*^1–3^. These diseases can lead to substantial yield losses. Egypt lies within the stripe rust epidemic zone, where yield losses due to severe outbreaks of yellow rust have been reported to reach up to 100%. Identifying the prevalent race or races of rust pathogens in a given location is important for effective breeding programs and disease management strategies^4,5^.

Wheat rust pathogens have a remarkable ability to overcome host resistance and adapt to changing climatic conditions, enabling them to persist across diverse regions worldwide. This adaptability is driven by mechanisms such as mutation, migration, and both somatic and sexual hybridization. Consequently, many previously effective resistance genes have lost their efficacy. However, some resistance genes continue to provide durable protection against a broad spectrum of pathogen races, despite the emergence of new virulent strains^6–8^.

Molecular markers are widely used to study genetic diversity, population structure, evolutionary relationships, and differences in pathogenic types of fungal plant pathogens, as well as their virulence. With advances in sequencing technology, single nucleotide polymorphisms (SNPs) have become an important type of molecular marker^9^. SNPs can detect variations at a single nucleotide level across both coding and non-coding regions of the genome. Compared to microsatellite markers, SNPs are more reliable. Microsatellites can produce uncertain outcomes due to mutations and the presence of null alleles. In contrast, SNP markers focus on single base changes and are analyzed using simpler mutation models. SNP markers also better signify the whole genome and display lower variation between loci SNP markers are increasingly used in studies of plant pathogen populations^10,11^.

Rust management through the application of biocontrol agents is one of the most widely used strategies for increasing grain production worldwide. Recently, this approach has gained significant attention as an alternative to synthetic fungicides due to its environmentally friendly nature and effective plant disease control mechanisms^12,13^. Numerous studies have demonstrated the potential of *Trichoderma* and Gliocladium species as biocontrol agents against a wide range of plant diseases. Among these, *Trichoderma* spp. have received particular attention and are considered promising candidates for future applications. Over the past five decades, extensive research has focused on the ability of *Trichoderma* spp. to reduce the incidence of diseases caused by plant pathogens. These fungi employ multiple mechanisms of action, including lysis, competition, mycoparasitism, antibiosis, and plant growth promotion. It is widely accepted that their successful antagonistic activity results from a combination of these mechanisms^14,15^.

Another effective biocontrol agent capable of reducing plant disease severity is the alga *Sargassum latifolium*, which is commonly found in diverse aquatic habitats and was collected from Egypt’s Red Sea coast^16,17^. This alga produces a wide range of secondary metabolites with various biological activities, including antiviral, antibacterial, and antifungal properties. Additionally, it synthesizes several bioactive compounds such as meroterpenoids, sterols, glycolipids, fucoidans, and phlorotannins, which contribute to its biocontrol potential^18^.

Recent advances have highlighted the potential of nanochitosan (chitosan nanoparticles) as an effective biocontrol agent, owing to its biocompatibility, biodegradability, broad-spectrum biological activity, and environmental safety. Previous studies have demonstrated its efficacy in managing several plant diseases including wheat rusts^19,20^.

Despite this progress, the molecular mechanisms underlying the pathogenesis of *Puccinia striiformis* f. sp*. tritici* remain insufficiently understood. Consequently, computational approaches have emerged as valuable tools for elucidating pathogen–host interactions. Among these, molecular docking analysis is particularly significant, as it provides detailed atomistic insights into molecular recognition by predicting the binding affinity and interaction modes between ligands and target proteins. In this respect, the effector protein *Pst11215* from *P. striiformis* f. sp. *tritici* plays a critical role in pathogenicity. It interacts with the wheat voltage-dependent anion channel TaVDAC1, a negative regulator of plant resistance, and promotes its ubiquitination via the E3 ligase TaVDIP1. This interaction suppresses the accumulation of reactive oxygen species (ROS) in mitochondria, thereby enhancing host susceptibility. Due to its key role in weakening wheat defense, Pst11215 was selected as a molecular target for structure-based virtual screening of potential inhibitors^21^.

To date, no studies have investigated the modes of action of *S. latifolium*, *T. harzianum*, and chitosan nanoparticles against *P. striiformis* f. sp. *tritici* using molecular docking analysis.

The objectives of this study are to: identify physiological races of virulence; analyze the molecular diversity within *P. striiformis* f. sp*. tritici* populations; select resistant wheat lines for future breeding programs; and evaluate the effectiveness of biological control agents. Additionally, molecular docking analysis will be conducted to identify potential antifungal ligands from *S. latifolium*, *T. harzianum* and chitosan nanoparticles using homology models of Pst proteins, in order to better understand ligand–protein interactions that may inhibit the infection process.

## Materials and methods

### Identification of *P. striiformis* f. sp.* tritici* Isolates using SNP markers

Urediniospores of rust races were collected from infected leaves 15 days post-infection. Genomic DNA was extracted from the dried urediniospores using the GeneJET Genomic DNA Purification Kit (Thermo Scientific, Lithuania, USA). DNA quality and concentration were assessed using a Nanodrop spectrophotometer. The single nucleotide sequence of the SNP gene of the studied isolates genome was identified with the specific SNP primer “ubiquitin-activating enzyme E1” (Uba1715S: ACCCAAACCACGGAACCC and Uba2088A: TCGCTCCAGCACCAACTA)^10^. PCR amplification was performed by using thermal cycler for PCR (Bio-Rad T100, Hercules, CA, USA). The amplification of PCR was programmed at 94 °C for 5 min as an initial denaturation cycle, followed by 35 cycles. Each cycle comprised (94 °C for 25 s, 59 °C for 25 s, then 72 °C for 45 s) with a final extension at 72 °C for 5 min. The amplification products were determined by electrophoresis in a 1.5% agarose gel. The PCR products were purified by using a gel extraction kit; then, the purified PCR products were sent for sequencing by Macrogen (Seoul, Korea). Then, the single nucleotide sequences of the SNP gene of the studied fungal isolates genomes were compared with similar sequences of strains of *P. striiformis* in the NCBI database, as established through the use of the Basic Local Alignment Search Tool (BLAST), which is identified on the website of the NCBI (https://blast.ncbi.nlm.nih.gov, 2020). Then, phylogenetic trees were constructed by using the MEGA 6 software program (https://mega.software.informer.com/6.0/, 2020). The phylogenetic analyses were carried out by using the maximum likelihood tree method. The tree distance was calculated using the maximum composite likelihood method.

### Greenhouse and field experiments

#### Plant materials, experimental design, greenhouse and field conditions

Avocet *S* near-isogenic lines (NILs), along with the European and World stripe rust differential sets and additional European wheat varieties carrying specific stripe rust resistance genes, were included in the differential set^22^ as shown in Table 1. A survey of stripe rust was conducted across four provinces: Kafr El-Sheikh (31°06′25.20″N, 30°56′26.99″E), Dakahlia, Gharbia (31°06′–31°50′E and 30°10′12″–31°31′48″N), and Damietta (31°04′N and 31°10′–31°30′E). The survey was carried out during the 2022–2023 and 2023–2024 wheat growing seasons and included approximately 50 wheat fields (Table 2). A total of 247 samples (leaves and spikes) were randomly collected from bread wheat fields, as presented in Table 2. The samples were placed in Petri dishes containing a double layer of moist filter paper and incubated at room temperature for 3 h to detect the presence of stripe rust infection. Subsequently, the samples were used to inoculate 10-day-old seedlings of the susceptible wheat cultivar “Morocco,” which had been treated with malachite hydrazine (MHP). Stripe rust urediniospores produced on the susceptible cultivar were collected 14–15 days after inoculation and stored at − 80 °C until further use.

**Table 1:** Differential cultivars used in the Central Western Asia Yellow Rust Trap Nursery.

Differential cultivars	Resistance gene/genes (Yr)
GI. World differential set^a^	
Chinese 166	*1*
Lee	*7,*+
Heines Kolben	*6,*+
Vilmorin 23	*3,*+
Moro	*10*
Stubes Dikkopf	*Sd,25,*+
Suwon92/Omar	*Su*
Clement	*9,2,*+
* T. spelta album*	*5*
GII. European Differential set^a^	
Hybrid 46	*4,*+
Reichersberg 42	*7,*+
Heines Peko	*2,6,25,*+
Nord Deprez	*3Nd*
Compare	*8,*+
Carstens V	*32,25,*+
Spaldings Prolific	*Sp,25,*+
Heines VII	*2,25,*+
GIII. Supplemental Differential set^a^	
Sonalika	*2, A*
Anza	*18,A*
Fed./Kavkaz	*9*
Seri 82	*9, 7*
Opata	*27,18*^*a*^*,*+
TP 981	*25,*+
Morocco	Check
GIV. Avocet Near Isogenic Lines^b^	
Avocet S	*AvS*
Avocet A	*A*
Avocet/*Yr1*	*1,18,AvS*
Avocet/*Yr5*	*5,18,AvS*
Avocet/*Yr6*	*6, AvS*
Avocet/*Yr7*	*7, AvS*
Avocet/*Yr8*	*8*
Avocet/*Yr9*	*9, AvS*
Avocet/*Yr10*	*10,18,AvS*
* YrSk*/3*AvocetS	*29*
* Yr11*/3*Avocet S	*11*
* Yr12*/3*Avocet S	*12*
* Yr15*/6*Avocet S	*15,18,AvS*
* Yr17*/6*Avocet S	*17,AvS*
* Yr18*/6*Avocet S	*18*
Avocet/*Yr27*	*27,AvS*
Avocet/*Yr32*	*32,AvS*
* YrSp*/6*AvocetS	*Sp,18,Avs*
GV. Common Cultivars	
Cook	*APR*
Corella	*6,7*
Oxley	*6,APR*
Kalyansona	*2,*+
Jupateco 'R'	*18*+
Jupateco 'S'	–
Crankbrook	–

^a^Recurrent parent (Avocet), ^b^Near Isogenic Lines (NILs) derived from Avocet containing resistance genes.

**Table 2:** Survey of yellow rust samples, number of samples, isolates and races of stripe rust during 2022/2023 and 203/24 seasons.

Governorate	2022/2023				2023/2024			
	Sample	Isolate	Race		Sample	Isolate	Race	
Kafr El-Sheikh	**42**	**33**	**9**	(9)*	**35**	**30**	**12**	**(12)**
Kafr El-Sheikh	11		2	2	9		3	3
Sidi Ghazi	6		2	2	5		2	2
Masir	5		1	1	3		2	2
Motobas	7		2	2	5		2	2
Qallin	8		1	1	6		2	2
El manshiya	5		1	1	7		1	1
Gharbia	**35**	**20**	**9**	**(3)**	**35**	**23**	**9**	**(8)**
Tanta	8		1	1	6		1	1
Qatour	5		2	–	5		1	1
Santa	9		1	1	8		2	2
Mahalla	3		1	–	4		1	1
Basyoun	4		1	1	5		1	1
Samannoud	3		1	–	3		1	1
Zaftaa	3		2	–	4		2	1
Dakahlia	**40**	**30**	**9**	**(7)**	**25**	**19**	**7**	**(6)**
Faraskour	6		3	2	7		2	2
El Serw	4		2	2	5		2	1
Ras El Bar	3		2	2	3		1	1
Kafr El Battikh	–		–	–	2		1	1
El Zarqa	2		2	1	1		1	1
Damietta	**15**	**15**	**7**	**(4)**	**20**	**14**	**6**	**(2)**
Total	**132**	**98**	**34**	**(23)**	**115**	**86**	**34**	**(28)**

Significant values are in bold.

*Races without recurrence.

Four seeds of each stripe rust differential genotype were seeded at a rate of four entries per plastic pot (genotype per each corner of square plastic pot). The stripe rust isolates’ stored urediniospores were combined 1:10 with talc powder (SOLTROL 170) and subsequently infected the genotypes at the seedling stage. Following the development of urediniospores, the seedlings were rubbed differently, incubated for 24 hs at 9 ± 1 °C in a dew chamber, and then moved into a greenhouse (15 ± 2 °C). Seedlings were assessed using a 0–9 scale outlined by^23^ following 15–17 days of inoculation. According to^22^, genotypes with infection types (IT) ranging from 0 to 6 were regarded as low infection types and showed no virulence, but those with (IT) ranging from 7 to 9 were regarded as high infection types and showed virulence. Virulence and avirulence^22^, which correlate to genes that resist stripe rust, are indicated by symbols. During two growing seasons in 2023 and 2024, the current studies were conducted in the stripe rust greenhouse and experimental farm at Sakha Agriculture Research Station, Plant Pathology Research Institute (PPRI), Agricultural Research Center (ARC).

Twenty wheat cultivars and 52 isogenic lines obtained from CIMMYT were evaluated for their response to the stripe rust pathogen at both seedling and adult plant stages (artificial inoculation) (Tables 3, 4, 5). Field experiments were conducted at Sakha Agricultural Research Station during the 2022–2023 and 2023–2024 wheat growing seasons. The experimental design was a randomized complete block design (RCBD) with three replicates for each genotype. Each genotype was sown in two rows, each 3 m long, with 30 cm spacing between rows and 20 cm between plants within rows. To ensure disease spread, the susceptible wheat cultivar “Morocco” was planted as spreader rows to facilitate the distribution of stripe rust inoculum.

**Table 3:** List of the tested wheat genotypes used in this study with their pedigree.

No	Genotypes	Pedigree
1	Sakha94	CMBW90Y3180-0TOPM-3Y-010 M-010 M-010Y-10 M-015Y-0Y-0AP-0S
2	Sakha95	CMA01Y00158S-040POY-040 M-030ZTM-040SY-26 M-0Y-0SY-0S
3	Gemmeiza7	CMH 74 A.630/SX // SERI 82 /3/ AGENT GM 4611-2GM-3GM-1GM-0GM
4	Gemmeiza9	GM 4583-5GM-1GM-0GM
5	Gemmeiza10	CG5820-3GM-1GM-2GM-0GM
6	Gemmeiza11	GM7892-2GM-1GM-2GM-1GM-0GM
7	Gemmeiza12	CMSS97Y00227S-5Y-010 M-010Y-010 M-2Y-1 M-0Y-0GM
8	Sids1	HD2172/Pavon‘‘S’’//1158.57/Maya74‘‘S’’SD46-4Sd-2SD-1SD-0SD
9	Sids12	SD7096-4SD-1SD-1SD-0SD
10	Sids13	KAUZ"S”//TSI/SNB"S”. ICW94-0375-4AP-2AP-030AP-0APS-3AP-0APS-050AP-0AP-0SD
11	Sids14	Bow’’s’’/Vee’’s’’//Bow’s’/Tsi/3/BANI SUEF 1 SD293-1SD-2SD-4SD-0SD
12	Misr1	CMSS00Y01881T-050 M-030Y-030 M-030WGY-33 M-0Y-0S
13	Misr2	CMSS96M03611S-M-010SY-010 M-010SY-8 M-0Y-0S
14	Misr3	CMSS06Y00582T099TOPM-099Y-099ZTM-009Y-099 M-10WGY-0B-0EGY
15	Misr4	NS-732/HER/3/PRL/SARA/TSI/VEE#5/4/FRET2/5/Wheat/SOKOLL
16	Shandaweel1	CMSS93B00567S-72Y-010 M-010Y-010 M-3Y-0 M-0HTY-0SH
17	Shandaweel2	QUAIU/5/FRET2*2/4/SNI/TRAP#1/3/KAUZ*2/TRAP//KAUZ. CMSS06B00109S-0Y-099ZTM-099NJ-13WGY-0B-0SH
18	Nubaria 2	FRET2*2/4/SNI/TRAP#1/3/KAUZ*2/TRAP//KAUZ*2/5/BOW/ URES//2*WEAVER/3/CROC_1/AE.SQUARROSA (213)//PGO. CGSS05B00144T-099TOPY-099 M-099NJ-099NJ-7WGY-0B-5Y-0B0NUB
19	Giza168	CM93046-8 M-0Y-0 M-2Y-0B-0SH
20	Morocco	NA

**Table 4:** Yellow rust infection types (IT) and disease severity (DS) for 20 cultivars released in Egypt against yellow rust races at seedling and adult plant stages during 2023 and 2024 seasons.

Cultivare	Race/seedling (IT)					Adult reaction (DS)	
	111E255	191E255	175E255	239E255	246E175	2023	2024
Sakha 94	7	7	7	8	9	10S	5 S
Sakha 95	8	6	5	7	9	20S	10S
Gem.7	7	7	6	8	8	40S	20S
Gem.9	8	7	8	9	9	30S	20S
Gem.10	7	7	8	6	8	5MS	TRS
Gem.11	7	7	8	7	9	80S	70S
Gem.12	8	7	8	7	9	30S	30S
Sids 1	8	8	7	8	9	20S	10S
Sida 12	8	5	7	8	8	60S	90S
Sids 13	8	8	9	8	9	0	5MS
Sdis 14	8	8	9	8	9	10S	30S
Misr 1	7	8	8	7	9	40S	60S
Misr 2	4	7	7	8	9	60S	50S
Misr 3	1	4	4	7	9	Tr.MR	5R
Misr 4	4	1	4	0	5	0	0
Shandwell.1	7	7	5	6	8	60S	20S
Shandwell.2	7	7	7	5	8	20MS	10MS
Nubaria 2	4	6	2	5	7	0	5MS
Giza 168	3	4	5	4	5	Tr.MR	5MS
Morocco	8	8	9	8	9	80S	90S

**Table 5:** Yellow rust infection types (IT) and disease severity (DS) for 52 CIMMYT wheat monogenic lines against yellow rust races at seedling and adult plant stages during 2023 and 2024 seasons.

Yr gene/source	Infection type on seedlings					Disease severity and infection response	
	111E255	191E255	175E255	239E255	246E175	2023	2024
Avocet-YRA	8	9	9	9	9	80S	90S
Avocet + YRA	8	9	8	7	9	30S	80S
YR1/6*AOC	8	9	8	8	9	40S	50S
SIETE CERROS T66	9	8	6	7	7	70S	60S
TATARA	0	0	2	0	0	0	TrS
YR5/6*AOC	2	2	2	2	3	0	0
YR6/6*AOC	8	7	8	3	7	80S	90S
YR7/6*AOC	5	4	6	7	6	90S	70S
YR8/6*AOC	2	0	0	3	0	0	TrS
YR9/6*AOC	4	5	4	8	8	80S	90S
YR10/6*AOC	8	9	8	9	8	10S	5 S
YR15/6*AOC	2	2	0	0	2	0	0
YR17/6*AOC	4	6	4	4	6	30S	30S
YR18/3*AOC	9	8	8	8	8	60S	50S
YR24/3*AOC	3	4	3	3	2	40S	30S
YR26/3*AOC	4	3	0	3	5	30MS	50S
YR27/6*AOC	8	6	9	9	9	20MR	10MR
YRSP/6*AOC	2	4	2	2	6	0	0
PAVON F 76	8	4	8	8	9	30S	20S
SERI M82	8	8	8	8	8	70S	40S
OPATA M85	9	9	8	8	7	TrMR	5MR
SUPER KAUZ	8	8	9	4	9	10MR	10MR
YRCV/6*AOC	6	5	5	4	6	70S	80S
PBW343	8	9	9	3	7	60S	50S
AOC-YR*3/3ALTAR84/ AE.SQ//OPATA	5	4	5	9	8	10S	5 S
AOC-YR*3//LALBMONO1*4/PVN	7	6	7	6	9	60S	40S
AOC-YR*3/PASTOR	5	5	5	7	9	100S	100S
POLLMER	8	9	9	9	7	70S	4MR
PASTOR	8	8	9	4	9	70S	90S
REBECA F2000	8	9	8	9	8	60S	20S
FRANCOLIN#1	8	8	8	7	8	80S	70S
AOC-YR/QUAIU#3	8	8	9	6	9	80S	90S
OPATA/PASTOR	8	7	8	7	7	0	5R
OPATA/PASTOR	8	7	8	7	7	0	0
AOC-YR/QUAIU#3	8	9	9	8	9	30S	80S
M10(MUTATED C-306)/AOC-YR	8	5	5	6	6	30MR	60S
CHUAN NONG 19	9	8	9	8	8	0	0
IRAGI	9	9	9	9	7	Tr.R	Tr.S
KOELZ W 11,192:AE	8	5	5	6	8	30S	50S
PBW343/KKU	8	6	7	6	9	0	0
AOC-YR*3//LALBMONO1*4/PVN	7	7	7	9	8	30S	90S
YR33	9	5	9	6	9	TrMR	0
YR34	9	6	6	9	9	30MR	50S
YR35 98M71	6	6	2	5	8	70S	80S
YR37	6	6	6	7	9	30S	0
YR4PL	7	5	6	9	7	TrR	TrR
YR51	9	4	9	8	6	TrR	5R
YR54	9	9	7	8	9	70S	80S
YR57	8	9	9	8	7	0	0
YRKK	7	6	6	6	9	0	0
YRALd	4	2	0	3	2	0	1 0S

### Disease assessment

The rust reaction data of the mature plant was graded as plant response and rust severity when rust symptoms were fully established, almost at the early dough stage. According to^24^ five infection types (ITs) represents the plant response. Modified Cobb’s scale was used to score the rust data four times for the disease severity as a percentage covering of leaves with rust pustules at weekly intervals once the spreader plants had 50% infection.

### Bio control assay

#### Preparation of culture filtrates from biocontrol agents and aqueous extracts of *Sargassum latifolium* and Chitosan nanoparticles

*Trichoderma harzianum* was cultivated in conical flasks containing 200 ml of potato dextrose broth media (PDB, Difco). The flasks were incubated for ten days on a rotary shaker (150 r.p.m.) at 28 ± 2 °C. After that the broth media were filtered through muslin cloths and the filtrates were subsequently centrifuged in an electrical centrifuge at 10,000 r.p.m. for ten minutes. The culture filtrate was sterilized using a Seitz filter then kept for further use^13^. *Sargassum latifolium* was acquired from the Botany Department, Faculty of Science, Tanta University, Tanta, Egypt. Firstly, *S. latifolium* was washed several times with tap water and distilled H₂O. After draining the remaining water, the seaweed was spread out on blotting paper and allowed to dry in the shade at 25 °C before being ground into a powder. Dry plastic bags are used to keep the ground-dried algae components until they are needed again^25^. Then, 1g of algal powder and 100mL of distilled water were combined to create the aqueous extract, which was then continuously shaken for 15 min at 60 °C using a glass stirring rod. The resulting extract was filtered using Whatman No. 1 filter paper, and the filtrate was utilized as a treatment^25^. In addition, Chitosan nanoparticles were synthesized and characterized in accordance with^26^ and utilized in this investigation. A nano-chitosan concentration of 150 μg mL^−1^ (150 ppm) was utilized.

#### Biological control of stripe rust disease

The biopotential efficiency of three biological treatments—*S. latifolium*, *T. harzianum*, and chitosan nanoparticles were evaluated against wheat stripe rust in the field during 2023/2024 growing season. The wheat cultivar (cv. Misr2) was established in experimental plots in three rows, each 3 m long and split 30 cm apart. 15 g of grains were spread throughout each row. Each plot had a distribution area surrounding by a Morocco wheat type, which is susceptible. All agricultural practices were carried out in accordance with the standard recommendations applied in commercial wheat fields in Egypt. Surface irrigation was performed every 25 days depending on climatic conditions and soil type, with planting on raised beds to enhance water use efficiency. Single superphosphate fertilizer was applied at a rate of 100 kg/feddan prior to sowing, and ammonium nitrate was added at a total rate of 75 nitrogen units per feddan, divided into three doses: at sowing, after the first irrigation and after the second irrigation. Selective herbicides were used during the early growth stages to control both broadleaf and grassy weeds. These practices aimed to provide optimal growing conditions for the plants and ensure reliable assessment of the studied cultivar resistance to yellow rust^27^. Using a hand sprayer until the plants were completely moist, the biological treatments were given twice throughout the seventh to eighth growth stage. The first spray was administered one day prior to the pathogen inoculation, and the second one was sprayed around fifteen days after the first one, at the onset of the infection (3%) and at that point. To compare, 0.25 ml L^−1^ of the fungicide PROPICONAZOLE 25% was utilized. We sprayed distilled water on the untreated control cultivars.

### Molecular docking analysis and computational analysis

Molecular docking of the tested biological treatments compounds with essential proteins involved in *P. striiformis* virulence Pst11215 was analysis using the AutoDock Vina software^28^ to define the interactions between the target virulence proteins of *P. striiformis* and the ligand structures of the biological treatments compounds and to determine the direct effects of these compounds on the inhibition of *P. striiformis*.

The sequence of Pst11215-targeted protein was downloaded from NCBI (https://www.ncbi.nlm.nih.gov/) in the FASTA format with accession number (XM_047952639) to build binding models with a 3D structure by using the AlphaFold Protein Structure Prediction Server, a high-end deep-learning –driven algorithm for predicting high-confidence 3D models from FASTA sequences. The affinity minimization of the targeted protein was carried out using the 3DREFINE server (http://sysbio.rnet.missouri.edu/3Drefine/index.html).

To download targeted ligands of biological agents for molecular docking through online databases and scientific literature for identified bioactive compounds used by several researchers as presented in the Table (S1). While utilizing tools also used for ligands downloaded like PubChem (https://pubchem.ncbi.nlm.nih.gov/) in the SDF format, the Open Babel software (http://openbabel.org/wiki/Main_Page) was then used for the conversion into the MOL2 format. All 3D structures of the ligands were minimized in energy with Avogadro 1.2.0 Software^29^, utilizing the MMFF94 force field to generate optimized conformations appropriate for molecular docking experiments. Pre-docking, all water molecules and ligands were removed while hydrogen atoms were added to the target proteins. Interaction of *P. striiformis* protein built model (Pst11215) with the ligand structures of the biological treatments compounds. Docking of the targeted protein was carried out against the studied compounds by the aid of AutoDock Vina software^28^. The calculations of binding free energies were performed using scoring function of AutoDock Vina as element in its script. Following an exhaustive search, 100 poses were analyzed and the best scoring poses were selected to calculate the binding affinity of the ligands. In addition, Discovery Studio (https://www.discngine.com/discovery-studio) was used for the 2D structures of the ligands.

### Thousand kernel weight (TKW)

Thousand-kernel weight is one method of determining seed size indication of wheat grain yield. Thousand-kernel weight of each variety were chosen at random and weighed from each plot.

### Statistical analysis

The data were subjected to ANOVA using the IBM SPSS Statistical software program (version 25), followed by Duncan multiple range tests (*P* ≤ 0.05) to determine significant differences between treatments. A similarity matrix of all wheat cultivars were performed using phenotypic data, and disease severity analysis was used to construct a dendrogram using the weighted pair group method with the arithmetic means clustering method in the numerical taxonomy system (NTSYS-PC version 2.1).

## Results

### Molecular identification and phylogenetic analysis of *Puccinia striiformis* species

**Fig. 1:**
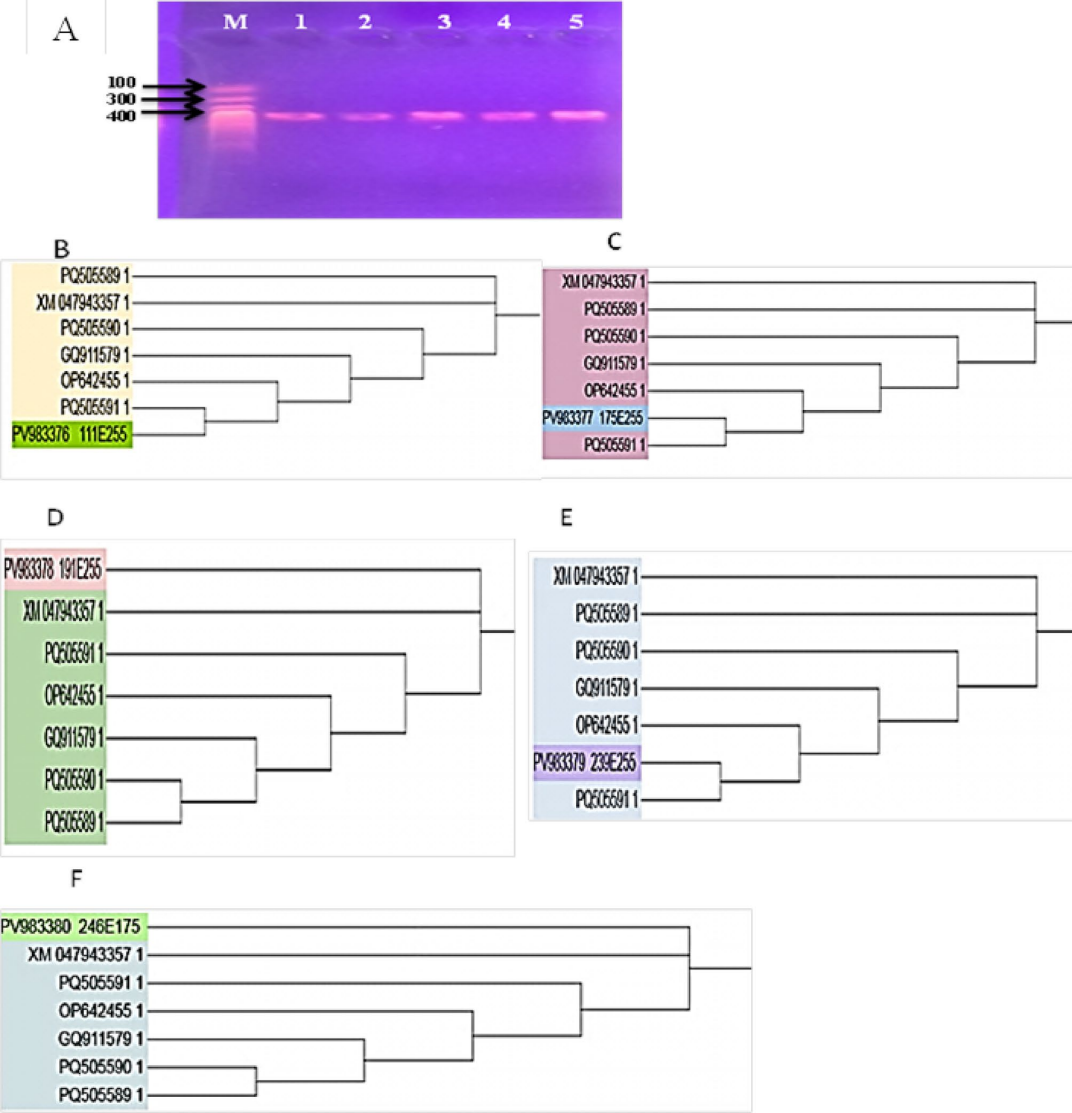
PCR product and phylogenetic tree of the five *P. striiformis* f. sp. tritici Isolates 1-5 (**B**–**F**). **A** PCR product of the isolates. M = Molecular marker, P. striiformis samples (1–5), **B**–**F** phylogenetic tree depend on the SNP gene sequences of five the isolates.

Results of molecular identification analyses of purified aggressive *P. striiformis* f. sp. *tritici* isolates (Fig. 1A–F) showed that the five PCR products of the single nucleotide sequence (SNP) were detected at 400 bp, (Fig. 1A). The sequence analysis of the fungal isolates results revealed 100% similarity to the *P. striiformis* f. sp*. tritici* SNP sequence, and they were deposited in the *GeneBank* under the five accession numbers PV983376, PV983377, PV983378, PV983379 and PV983380).

**Fig. 2:**
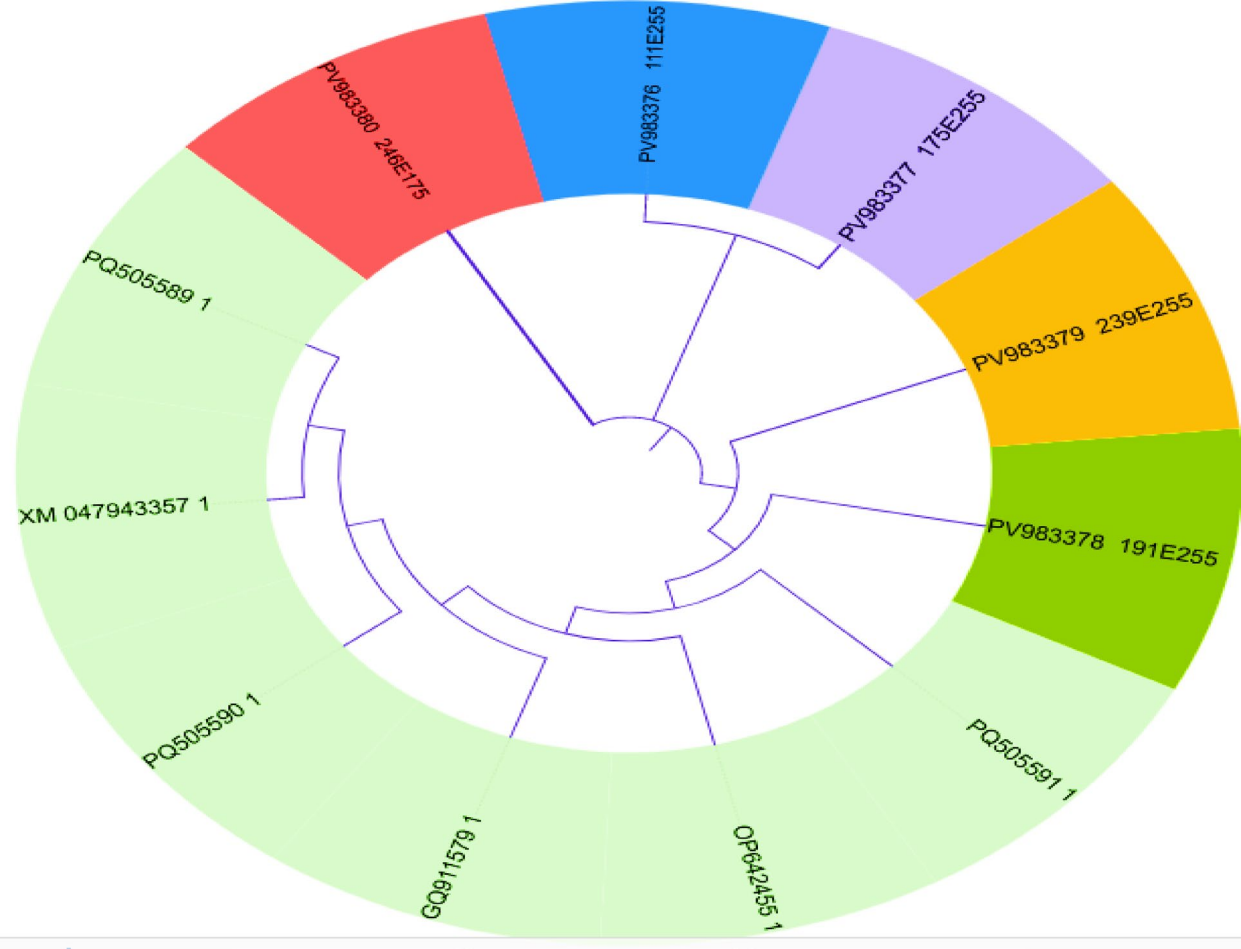
UPGMA dendrogram showing the relationship among *Puccinia striiformis* and novel aggressive races isolates.

The phylogenetic analysis demonstrated that the closeness of the genetic similarity between the studied five isolates i.e. 111E255, 175E255, 191E255, 239E255 and 246E175 having accession numbers PV983376, PV983377, PV983378, PV983379 and PV983380, respectively with others from around the world was mostly with strains of *P. striiformis* f. sp. *tritici* with different accession numbers (PQ505589.1, XM_047943357.1, OP642455.1 and GQ911579.1) in the database of the *GenBank* (Fig. 1). Therefore, *P. striiformis* was confirmed as the causal pathogen responsible for yellow rust disease in wheat in Egypt. The evolutionary relationships among *P. striiformis tritici* isolates are presented in Fig. 2. The phylogenetic analysis included five aggressive races with accession numbers PV983376, PV983377, PV983378, PV983379, and PV983380, and examined their relationships with other *P. striiformis* taxa. These relationships are illustrated as a phylogenetic tree, where branches represent evolutionary lineages over time and nodes indicate common ancestors. The optimal tree exhibited a total branch length of 29.50951280, with branch lengths expressed in the same units as the evolutionary distances used to construct the tree. The tree is drawn to scale. Understanding these evolutionary relationships is essential for studying the evolution and classification of *P. striiformis*. The phylogenetic analysis confirmed the genetic relationships between *P. striiformis* and the five newly identified aggressive races.

### A virulence survey, and germplasm screening

#### A virulence/virulence distribution of *P*. *striiformis* f. sp. *tritici (Pst* ) during 2023 and 2024 growing seasons

The present study highlights the distribution of virulence of Pst phenotypes detected for the Yrs single gene lines in Egypt as presented in Table 1. Field survey data confirmed that stripe rust remains a significant threat to widely cultivated wheat varieties, particularly Gemmeiza-11, Sids-12, Misr 1, and Misr 2, indicating their vulnerability at different levels. Kafr El-Sheikh Governorate recorded the highest number of samples in both seasons, with 21 identified strains. In contrast, Damietta Governorate showed the lowest number of detected strains, with 13 strains obtained from 35 samples (Table 2). A total of 20 races were identified during the 2023 season, 19 of which had been previously reported. In the 2024 season, 23 races were identified, including 19 previously reported races.

**Fig. 3:**
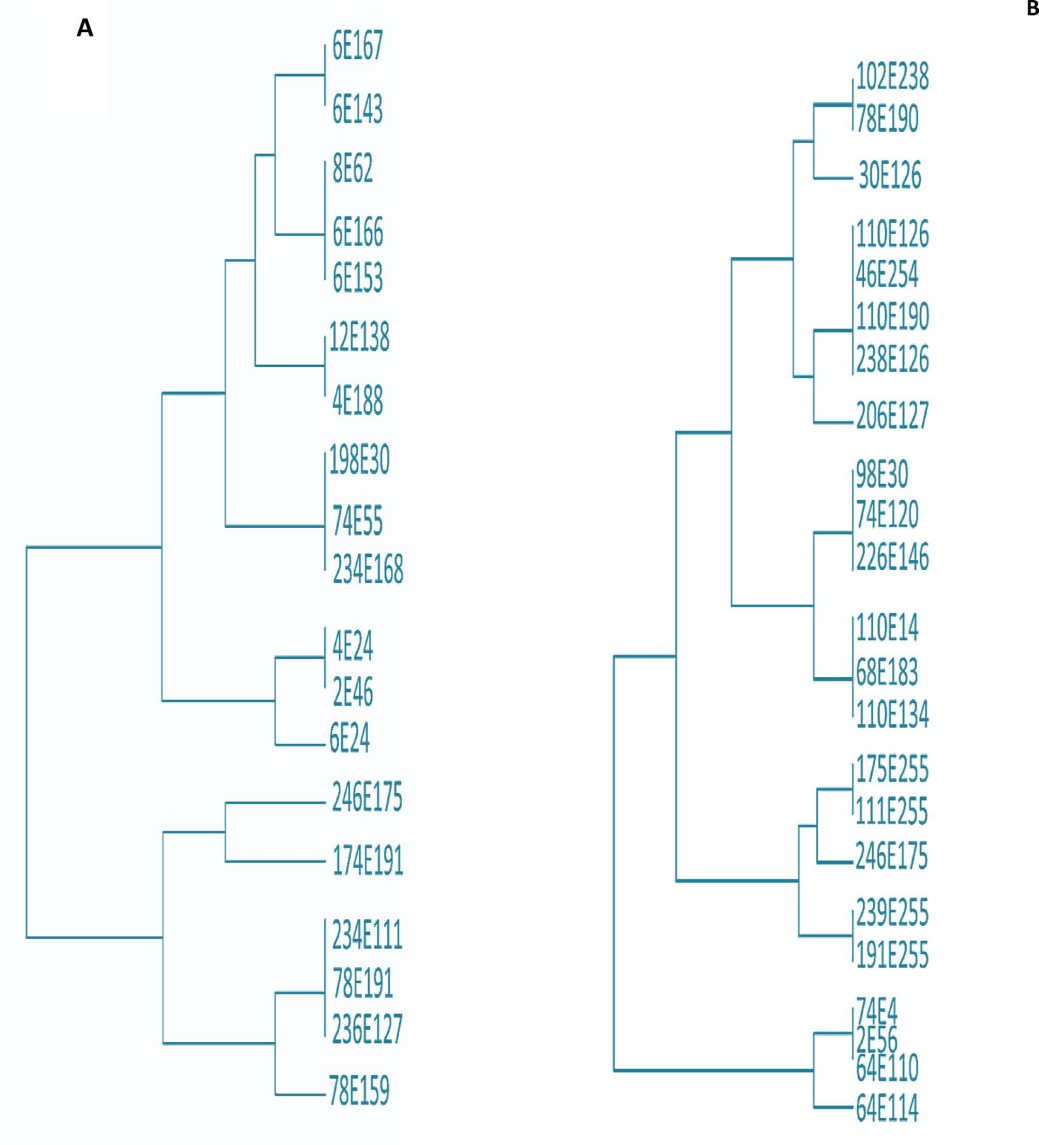
Dendrogram showing similarity index cluster analysis of identified *Puccinia striiformis* races based on their virulence during the 2023 (**A**) and 2024 (**B**) season

The investigation further revealed that five races were dominant at the seedling stage based on disease severity assessments conducted over the study period. Notably, four highly aggressive races were detected for the first time in Egypt during the second season (2024), while a fifth race was detected in both seasons on bread and durum wheat across multiple locations (Fig. 3A, B; Tables 6, 7).

**Table 6:** Virulence formulae of 20 identified *Puccinia striiformis* f. sp. *tritici* races during the 2023 season.

No	Source	Races	Virulence formulae	No of virulent genes	Race virulence %
1	Morocco	0E0	0	0	0
2	Gemmiza 11	6E24	7, 6, (3),8 /	4	23.52
3	Sdis 12	6E166	7, 6,(7),(6),CV,2/	6	35.29
4	Egased 22	74E55	7,3,SU,4,(7),(6),8,CV	8	47.05
5	Misr 2	78E191	7,6,3,SU,4,(7), 6, (3),8,CV, 2 /	11	64.70
6	Sids 14	198E30	7, 6, SU, 9, (7), (6), (3), 8/	8	47.05
7	Misr 1	8E62	3,(7),(6), (3),8,CV/	6	35.29
8	Misr 1	4E188	6,(6), (3), 8, 9, CV/	5	29.41
9	Sids 12	2E46	7, (6), (7),(3), CV/	3	17.64
10	Misr 1	12E138	6,3 ,(7),(3),2/	5	29.41
11	Misr 2	6E153	7,6,4,(3),8,2/	6	35.29
12	Gemmiza 11	6E143	7,6,4,(7),(6), (3),2/	7	41.17
13	Sids 12	4E24	6, (3), 8/	3	17.64
14	Misr 1	174E191	7,6,3,SD,9,2,4,(7),(6),(3),8,CV,2/	13	76.47
15	Sakha 95	78E159	7,6,3,SU,4,(7),(6),(3),8,2/	10	58.82
16	Morooco	234E168	7,3,SD, SU,9,(3),CV,2	8	47.05
17	Morooco	234E111	7,3,SD, SU,9,4,(7),(6),3,CV, SP	11	64.70
18	Gemmiza 11	236E127	6,3,SD, SU,9,4,(7),(6),(3),8,2	11	64.70
19	Sakha 95	246E175	1,7,6,3,10,SD,9,4,(7),(6),(3),8,CV, SP,2	15	88.23
20	Misr 2	6E167	7, 6,4,(7),(6),CV,2	7	41.14

**Table 7:** Wheat yellow rust races identify their frequency and virulence in Egypt during 2024 season.

No	Source	Races	Virulence	No of virulent genes	Race virulence %
1	Misr 1	2E56	7,(3),8,CV,	4	23.52
2	G. 11,	46E254	7,6,3,SD, (7),(6),(3),8,CV, SP,,2	11	64.70
3	sids 12,	191E255	7,6,3,9,10,SD,4,(7),(6),(3),8,CV, SP	13	76.47
4	sids 12	64E110	SU,8,CV, SP	4	23.52
5	Misr 1	64E114	SU, (7),8,CV, SP	5	29.41
6	G. 11	68E183	6,SU,4,(7),(6),8,CV,2	8	47.05
7	Misr 1	74E4	7,3,SU, (6)	4	23.52
8	G. 11	74E120	7,3,SU, (3),8,CV, SP	7	41.17
9	sids 14	78E190	7,6,3,SU, (7),(6),(3),8,CV,2	10	58.82
10	Misr 3	30E126	7,3,SU, (7),(6),(3),8,CV, SP	9	52.94
11	G. 11	98E30	7,SD, SU, (7),(6),(3),8	7	41.17
12	G. 11	102E238	7,6,SD, SU, (7),(6),(3),CV, SP,2	10	58.82
13	Misr 1	110E14	7,6,3,SD, SU, (7),(6),(3)	8	47.05
14	Misr 2	110E126	7,6,3,SD, SU, (7),(6),(3),8,CV, SP	11	64.70
15	G. 11	110E134	7,6,3,SD, SU, (7),(6),2	8	47.05
16	Misr 1	110E190	7,6,3,SD, SU, (7),(6),(3),8,CV,2	11	64.70
17	Morooco	111E255	1, 7,6,3,SD,4,SU, (7),(6),(3),8,CV, SP,2	14	82.85
18	Sids 12	175E255	1,7,6,3,SD,9,4,(7),(6),(3),8,CV, SP,2	14	82.85
19	Morooco	246E175	1,7,6,3,10,SD,9,4,(7),(6),(3),8,CV, SP,2	15	88.23
20	G. 11	206E127	7,6,3,SU,9,4,(7),(6),(3),8,CV, SP	12	70.58
21	Misr 2	226E146	7,SD, SU,9,(7),8,2	7	41.17
22	G.11	238E126	7,6,3,SD, SU,9,(7),(6),(3),CV, SP	11	64.70
23	Misr 1	239E255	7,6,10,SD, SU,9,4,(7),(6),(3),CV, SP,2/	13	76.47

Five races—111E255, 175E255, 191E255, 239E255, and 246E175—were documented for the first time and subsequently deposited in the *GenBank* database under accession numbers PV983376, PV983377, PV983378, PV983379, and PV983380, respectively (Table 8; Fig. 3).

**Table 8:** Five wheat yellow rust aggressive races identify their frequency and virulence in Egypt.

No	Source	Races	Virulence	No of virulent genes	Race virulence %	Accession no
1	Morooco	111E255	1,7,6,3,SD,4,SU, (7),(6),(3),8,CV, SP,2	14	82.85	PV983376
2	Sids 12	175E255	1,7,6,3,SD,9,4,(7),(6),(3),8,CV, SP,2	14	82.85	PV983377
3	Sids 12	191E255	7,6,3,9,10,SD,4,(7),(6),(3),8,CV, SP	13	76.47	PV983378
4	Misr1	239E255	7,6,10,SD, SU,9,4,(7),(6),(3),CV, SP,2/	13	76.47	PV983379
5	Morooco	246E175	1,7,6,3,10,SD,9,4,(7),(6),(3),8,CV, SP,2	15	88.23	PV983380

Five races—111E255, 175E255, 191E255, 239E255, and 246E175—were documented for the first time and subsequently deposited in the *GenBank* database under accession numbers PV983376, PV983377, PV983378, PV983379, and PV983380, respectively (Table 8; Fig. 3).

Among the resistance genes evaluated, Yr15 was identified as the most effective against Egyptian stripe rust, followed by Yr5, both showing resistance to a broad spectrum of races. In the 2024 season, virulence patterns ranged from 13 differential lines (for races 191E255 and 239E255) to 15 differential lines (for race 246E175). The most virulent race was 246E175 (88.23%), followed by races 111E255 and 175E255 (82.85%). Races 111E255 and 175E255 exhibited virulence on differential sets including 1, 7, 6, 3, SD, 4, SU, (7), (6), (3), 8, CV, SP, 2 and 1, 7, 6, 3, SD, 9, 4, (7), (6), (3), 8, CV, SP, 2, respectively (Table 7).

#### Response of some Egyptian wheat cultivars to stripe rust disease

**Fig. 4:**
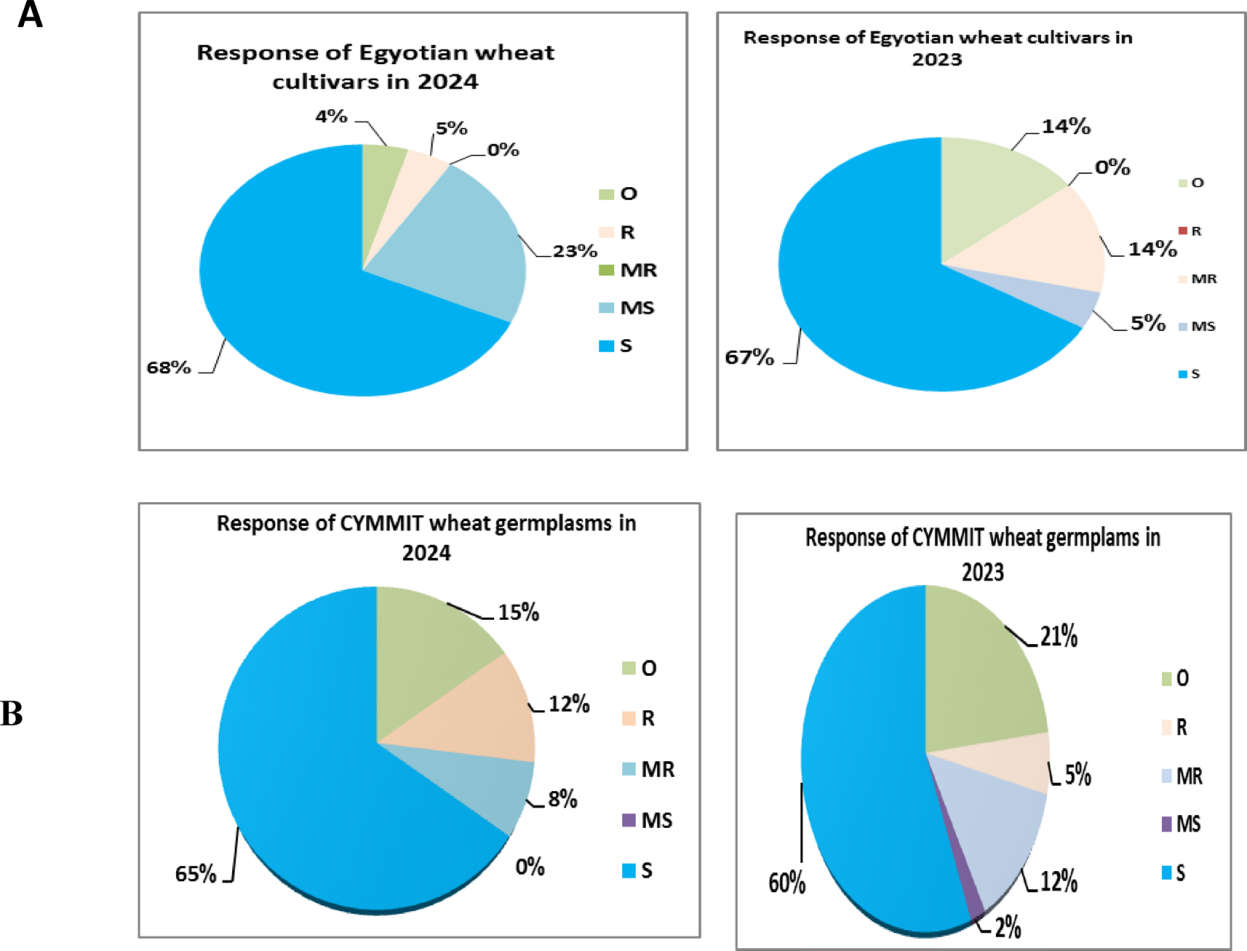
Graphical presentation of 20 Egyptian cultivars (**A**) and 52 CYMMET (**B**) against stripe rust at adult stage in two seasons 2023 and 24

Results presented in Table 4 and Fig. 4A revealed that the wheat cultivars Misr-4 and Giza 168 displayed resistance to most of the tested races under greenhouse conditions, whereas the majority of Egyptian wheat cultivars were highly susceptible at the seedling stage. During the two growing seasons, the adult plant response of Misr-4 (0) indicated near-immune resistance (NIR). In contrast, Misr-3 exhibited a reaction ranging from moderate resistance to resistance (MR–R) across both seasons. Additionally, the cultivars Giza 168 and Shandweel 2 (MR–MS) showed disease responses ranging from moderate resistance to moderate susceptibility. Meanwhile, Gemmeiza 10 was classified as moderately susceptible to susceptible throughout the study period. Sids 13 and Nubaria 2 demonstrated high levels of resistance in the first season; however, they became susceptible in the second season. Overall, the remaining evaluated wheat cultivars exhibited susceptible reactions to stripe rust under field conditions.

#### Responses of CIMMYT wheat genotypes carrying stripe rust resistance genes

**Fig. 5:**
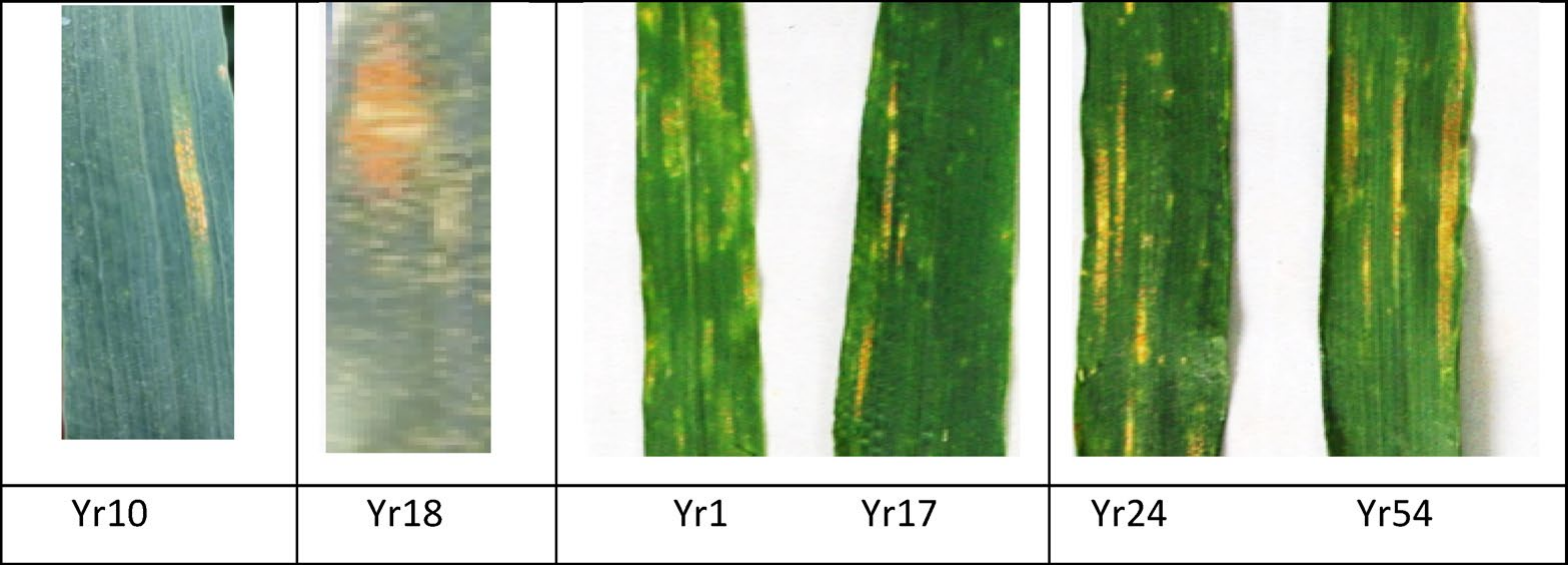
Responses of CYMMIT wheat genotypes carrying stripe rust resistance genes.

The result of this investigation revealed that ten of the germplasm—YR5, YR15, YRSP, OPATA/PASTOR, CHUAN NONG 19, PBW343/KKU, YR4PL, YR51, YR57, and YRKK—were clearly immune and resistant to the disease and may be advantageous to plant breeders’ efforts to fight this devastating disease on Egypt’s wheat crop. Throughout the two growing seasons, wheat genotypes displayed various rust responses. In most cases, the adult plant reactions were different from the seedling reactions as presented in Fig. 4B. Yr5, Yr15, and YrSp, at seedling and adult stages, exhibited the high resistance to all five races (Table 5, Fig. 5).

### Biocontrol evaluation

#### Potential of bio control agents to control wheat stripe rust

**Fig. 6:**
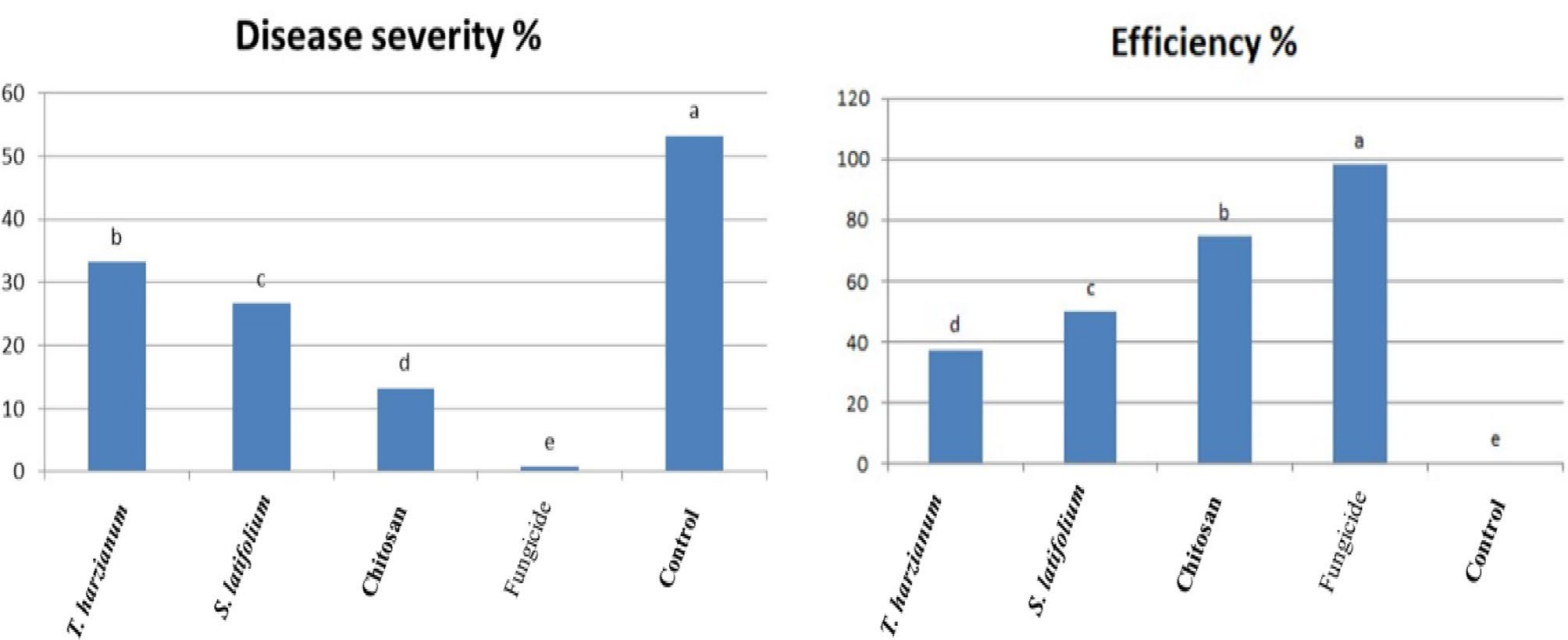
Efficiency of *S. latifolium*, *T. harzianum* and nano-chitosan on disease control of wheat stripe rust under field conditions during 2023/2024 growing season.” Different letters indicate significant differences between treatments (Duncan’s multiple range test at* P* < 0.05).

The antifungal efficacy of *S. latifolium*, *T. harzianum*, and chitosan nanoparticles against five aggressive *P. striiformis* isolates was initially assessed under greenhouse conditions, where high levels of control were recorded, and subsequently validated under field conditions. The antifungal activity of these treatments against the five aggressive *P. striiformis* isolates is illustrated in Fig. 6. In this study, the effects of different treatments on stripe rust–infected wheat were evaluated during the 2023/2024 growing season at the seventh growth stage (milk development stage). The results indicated that there was no significant difference in disease severity in the infected control plants that did not receive any biocontrol treatment. In contrast, all applied treatments significantly reduced the severity of stripe rust compared with the untreated control. Among all treatments, the fungicide “CRWAN” exhibited the highest efficacy. Regarding the biological treatments, chitosan nanoparticles showed the greatest reduction in disease severity, followed by *S. latifolium*, while *T. harzianum* demonstrated the lowest effectiveness. Overall, the findings confirm that biological treatments can significantly suppress the growth and severity of the five aggressive *P. striiformis* isolates under field conditions compared to the untreated 3.4 Molecular Docking Analysis and Computational analysis.

#### Effects of bio-control agents on the virulence proteins of *P. striiformis* using molecular docking analysis

**Fig. 7:**
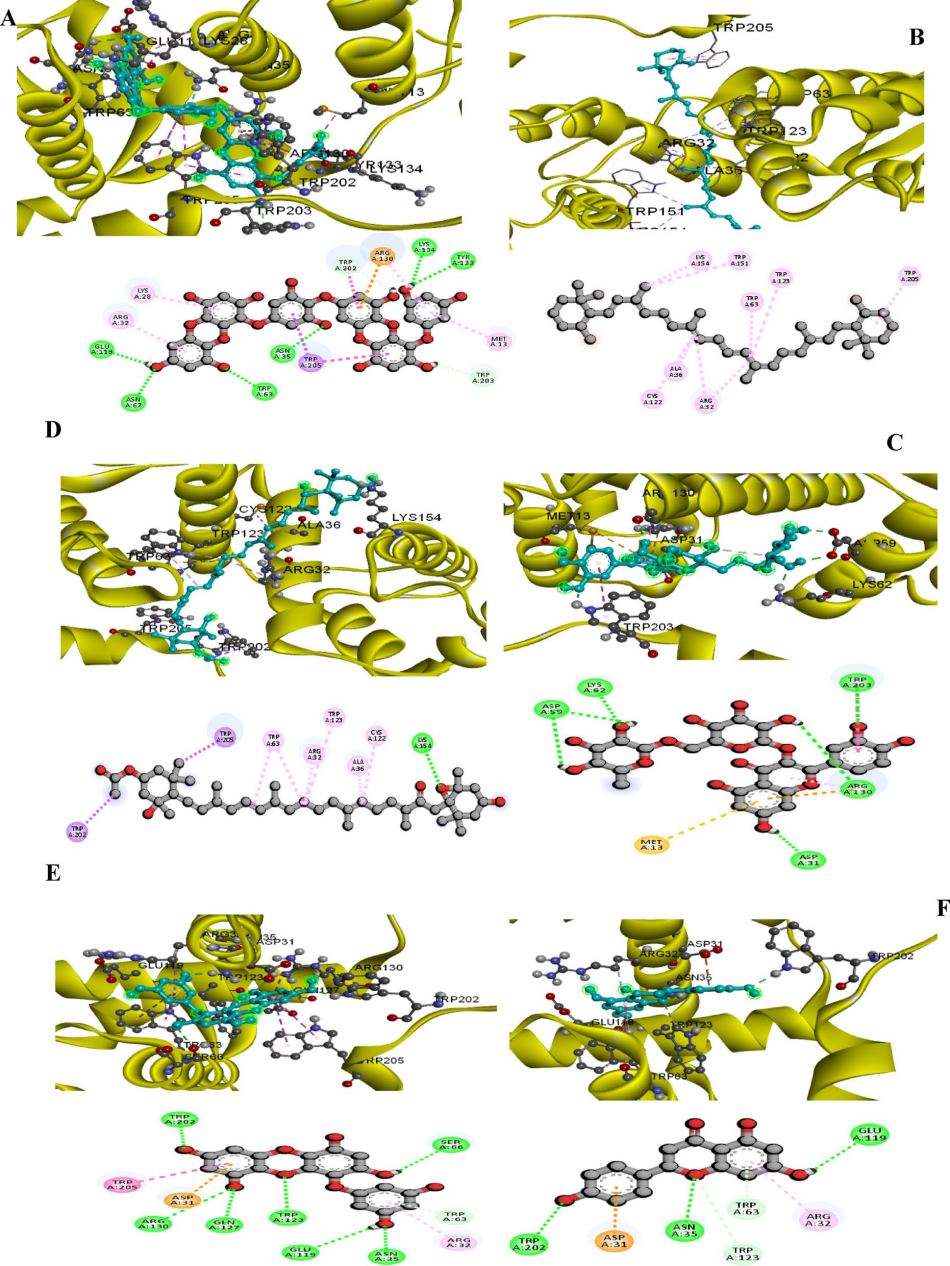
The 2D and 3D interaction diagrams of the binding of *Puccinia striiformis* f. sp. tritici. virulence protein (Pst11215) with the active ingredient of Dieckol (**A**) , Beta_Carotene (**B**) Fucoxanthin (**C**) Rutin (**D**) Eckol (**E**) and Naringenin (**F**) in *Sargassum latifolium*

**Fig. 8:**
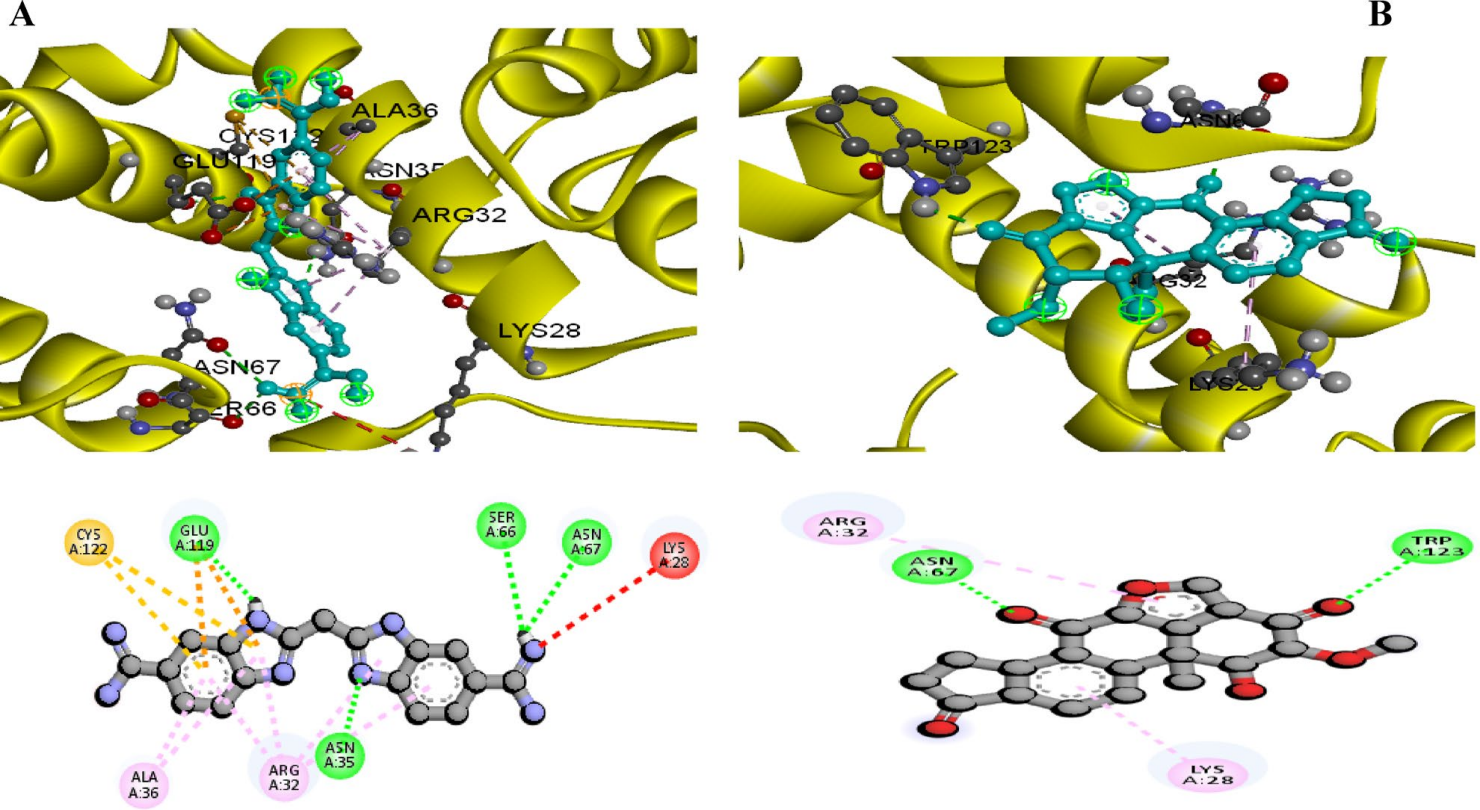
The 2D and 3D interaction diagrams of the binding of *Puccinia striiformis* f. sp. tritici. virulence protein (Pst11215) with the active ingredient of Chitinase (**A**) , and Viridin (**B**) in T. harzianum.

The *P. striiformis* proteins that were planned in relation to the active compounds present in *S. latifolium*, *T. harzianum* were docked as presented in Table 9, Figs. 7 and 8. This investigation was conducted to determine the ligands expected to block their activities and, hence, to gain a better explanation of their mode of action in controlling the pathogenicity of P. striiformis f. sp. tritici isolate. The vital proteins, i.e. *Pst11215* (Accession No.: XM_047952639) which have critical virulence pathways, were modeled. These proteins were the most vital candidates in the virulence of *P. striiformis* f. sp*. tritici*, as shown in Figs. 7 and 8. In these connections, Dieckol, Beta_Carotene, Fucoxanthin, Rutin ,Eckol , Naringenin compounds bound with the active sites of this protein with a binding affinity that ranged from − 10.9 to − 8 kcal/mol (Fig. 7). It is worth declaring that all of these compounds were present in the *in S. latifolium*. Moreover, the Chitinase , Viridin ligands had the highest score and bound with *Pst11215* protein with a binding energy that varied from − 9.5 to − 8.4 kcal/mol as presented in Fig. 8. However, these compounds were presented in *T. harzianum* extract. The model demonstrated the docking between the virulence proteins of *P. striiformis* and the ligands from the tested biological agent in order to clarify the mechanisms of the binding of the studied proteins in an attempt to understand their role in the inhibition of the *P. striiformis* pathogen.

**Table 9:** Effects of biocontrol agent, *S. latifolium*,* T. harzianum* on the *P. striiformis* f. sp. *tritici* virulence protein using the docking analysis.

Bio control agent	Protein\XYZ	Ligand name	Pubchem ID	Types of bond	Score
*Sargassum latifolium*	Pst11215 X = 1.455 Y = 0.129 Z = 2.155	Dieckol	3,008,868	Conventional Hydrogen Bond Carbon Hydrogen bonds	− 10.9
		Beta_Carotene	5,280,489	Pi-Donor Hydrogen Bond, Pi-Cation	− 9.3
		Fucoxanthin	5,281,239	Pi-Sigma Pi-Pi Stacked	− 9.2
		Rutin	5,280,805	Pi-Pi T-shaped, Pi-Alkyl	− 8.8
		Eckol	145,937	Alkyl	− 8.8
		Naringenin	439,246	Pi-Sulfur, Pi-Anion	− 8
*Trichoderma harzianum*		Chitinase	50,921,533	Conventional Hydrogen Bond	− 9.5
		Viridin	94,257	Pi-Anion Pi-Sulfur Pi-Alkyl	− 8.4

### Wheat yield production

**Fig. 9:**
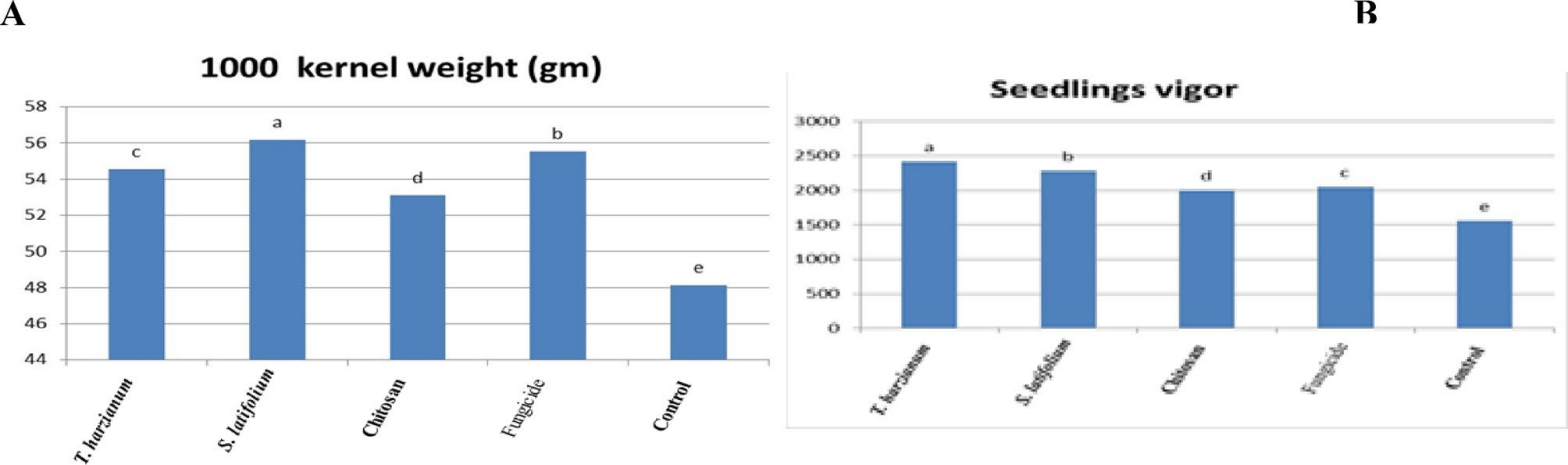
Effect of *T. harzianum*, *S. latifolium*, and Chitosan nanoparticles and CRWAN fungicide on thousands kernels weight (**A**) and seedling vigor (**B**) of wheat lines during 2023/2024 growing season. Different letters indicate significant differences between treatments (Duncan’s multiple range test at *P* < 0.05)

**Fig. 10:**
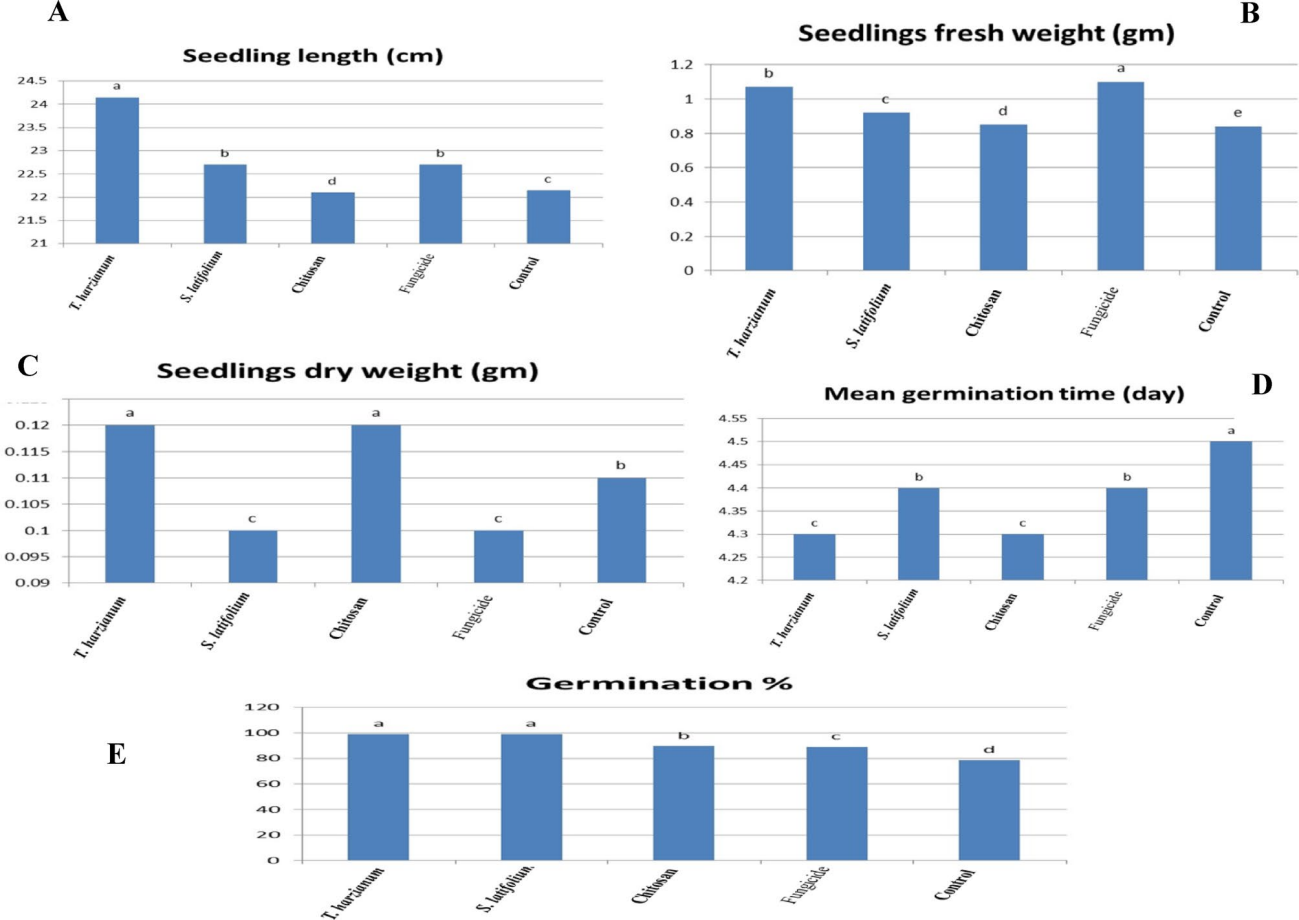
Effect of *T. harzianum*, *S. latifolium*, Chitosan nanoparticles and CRWAN fungicide on seedling length (**A**) , seedling fresh weight (**B**), seedling dry weight (**C**), mean germination time (**D**) and grain yield/plant, germination % (**E**) of wheat lines during 2023/2024 growing season. Different letters indicate significant differences between treatments (Duncan’s multiple range test at* P* < 0.05)

The results revealed that treated wheat with *T. harzianum*, *S. latifolium*, and chitosan nanoparticles had a considerably higher seed value per thousands of kernel weight (48.14g) than the untreated control (cv. Misr2) as presented in Fig. 9A. This suggests that the grains of genotypes treated with *T. harzianum*, *S. latifolium*, and chitosan nanoparticles are bolder and heavier than those treated with fungicide. Similarly, in *S. latifolium* (56.18), the plots with bolder and bigger seeds produced heavier grains than the control. In contrast, the large-size seeds of the wheat treated with *T. harzianu* and chitosan nanoparticles yielded the highest seed values (54.55) and (53.11), respectively. The results demonstrated in Fig. 9B also showed that there were significant differences between the treated wheat with respect of seedling vigor index. However, their interacting impact was statistically non-significant (*P* > 0.05). The results revealed that the quality of the seed straight impacts the increase in seed value, and if farmers are aware of this and exploit high-value seed, farmers may accomplish the favourite grain weight. Moreover, Fig. 10A–E shows that there were very important variations between the treated wheat in terms of seedling length (cm) , seedling fresh weight (gm), seedling dry weight (gm), mean germination time (day) and grain yield/plant, germination %,respectively.Treated plants produce the highest germination percentage, highest seedling length and seedling vigor index, heaviest seedling dry weight. The present investigation results demonstrate that wheat treated with bio control agent i.e. *T. harzianum, S. latifolium* and Chitosan Nanoparticles had a significant effect on the seed value. Subsequently, the results approved that the treated wheat had excellent agronomic and quality characteristics and produced large yields of wheat. Additionally, the treated wheat with bio control agent resistance to aggressive stripe rust in Egypt has improved wheat yields.

## Discussion

Stripe rust represents one of the most destructive biotic constraints, leading to significant economic losses in world wheat production^30^. The identification of prevailing races in specific regions is essential for the development of effective breeding programs and disease management strategies. However, the continuous evolution of rust pathogens, along with their ability to overcome resistance genes and adapt to changing climatic conditions, has rendered many resistance genes ineffective in several parts of the world. In the present study, five aggressive isolates of *P. striiformis* f. sp*. tritici* were successfully isolated and identified. In addition, the biocontrol potential of *S. latifolium* and *T. harzianum* against these isolates was evaluated, followed by an investigation of their modes of action using molecular docking analysis. Molecular approaches have been widely recognized as reliable alternatives to traditional fungal identification methods due to their high sensitivity, specificity, and rapidity. In *P. striiformis*, the ribosomal DNA single nucleotide polymorphism (SNP) region exhibits significant variability, making it a suitable target for molecular identification. The polymerase chain reaction (PCR) using specific primers that target this region have previously been shown to be an effective method for identifying pathogens^31^. The PCR-based SNP analysis in this study successfully classified the five isolates as *P. striiformis* races 111E255, 175E255, 191E255, 239E255, and 246E175, with accession numbers PV983376, PV983377, PV983378, PV983379, and PV983380, respectively. These findings are consistent with previous studies^32^ that utilized SNP markers within ribosomal DNA to differentiate among *P. striiformis* races. The results further confirm the high accuracy and sensitivity of SNP markers in detecting virulence diversity and monitoring race evolution within pathogen populations, highlighting their effectiveness as reliable tools for characterizing genetic variation and tracking evolutionary dynamics over time. Moreover, association mapping analysis is a powerful, high-resolution method for identifying molecular markers linked to avirulence genes, particularly when derived from polymorphic SNP regions. The selected Egyptian isolates exhibited diverse virulence profiles when tested on Yrs single-gene lines. Notably, none of the isolates showed virulence against Yr5, Yr15, or YrSP, indicating that these resistance genes remain effective. This observation aligns with previous reports highlighting the rarity of virulence against Yr5 and Yr15 worldwide^33^. However, the rapid spread of new virulent races, such as those overcoming Yr27 and the widely distributed Warrior race, emphasizes the urgent need to identify durable resistance sources^34,33^. Previous studies have demonstrated that the Yr18 gene contributes to partial, durable adult plant resistance, particularly when combined with other slow-rusting genes^35,36^. Similar resistance patterns have been reported in multiple regions, including China, Iran, Turkey, Iraq, North America, and Africa, where genes such as Yr5 have consistently shown high levels of resistance^37^. To the best of our knowledge, the presence of these five aggressive *P. striiformis* races has not been previously documented in Egypt, highlighting the importance of continuous surveillance and in-depth epidemiological studies. Field evaluations conducted during the 2023 and 2024 growing season’s revealed significant variation among wheat cultivars in their resistance to stripe rust across different locations. Cultivars Sids 13 and Nubaria 2 showed strong resistance during the first season but became more susceptible in the second season, suggesting possible influences from environmental conditions or changes in pathogen populations. In contrast, most of the remaining cultivars exhibited susceptible reactions under field conditions. These results are consistent with earlier studies^8^, which reported that only a limited number of commercial wheat cultivars maintain stable resistance to stripe rust across multiple growing seasons.

Rust management with biocontrol agents is one of the most popular methods for enhancing grain production worldwide. In recent years, biocontrol agents have increased a lot of attention as a potential replacement for synthetic fungicides that have plant disease-fighting properties. The antifungal potentials of biological control agents *S. latifolium* and *T. harzianum*, and chitosan nanoparticles were evaluated with respect to *P.striiformis*. The results showed that *S. latifolium* and *T. harzianum* efficiently inhibited the growth of the studied fungus with varying degrees of potency, consistent with previous reports highlighting their antifungal properties^38^. The results demonstrate, for the first time in Egypt, the use of *S. latifolium* as a biological agent on wheat. This alga is rich in nutrients, including proteins, lipids, carbohydrates, and trace minerals, which may contribute to the inhibition of yellow rust disease and the stimulation of wheat growth^39^. Consequently, wheat yield increased following treatment with *S. latifolium*. Moreover, chitosan nanoparticles reduced infection severity and the number of pustules, while extending latency and incubation periods and inhibiting spore germination. Previous studies^40^ have shown that chitosan nanoparticles can bind to microbial proteins, disrupt cell membrane integrity, and increase permeability, leading to strong antimicrobial effects, as well as inhibiting hyphal growth in plant pathogens. Anatomical analyses further revealed that chitosan nanoparticles increased the thickness of the leaf blade, mesophyll tissue, upper and lower epidermis, and the length and width of the midrib vascular bundle. In contrast, numerous urediniospores were observed on the upper epidermis in untreated control plants. In Egypt, nano-chitosan has already been reported to induce resistance to leaf rust^26^, whereas its effect on yellow rust disease has not yet been documented.

As substitutes for synthetic fungicides, these materials have recently drawn a lot of interest, and attempts have been made to use them in plant disease management methods. According to the current study, these components were just as effective as fungicides at controlling the wheat stripe rust disease. In an effort to make the change to more environmentally friendly practices, there is a search for viable candidates for bio-agents to supplement or replace synthetic pesticides due to the marked growth in interest in biological control. Computational molecular docking has emerged as a powerful tool for elucidating the mechanisms of action of antifungal compounds by identifying their interactions with key pathogenic proteins^41^. In the present study, molecular docking analysis was employed to investigate the ability of bioactive compounds derived from the tested biocontrol agents to bind specifically to essential protein targets involved in the infection process and growth of *Puccinia striiformis*.

The results revealed that several compounds identified in *S. latifolium*, including dieckol, β-carotene, fucoxanthin, rutin, eckol, and naringenin, exhibited strong binding affinities with the active sites of *P. striiformis* proteins associated with pathogenicity. Similarly, compounds derived from *T. harzianum*, such as chitinase and viridin, demonstrated effective binding interactions with the *Pst11215* protein. These interactions suggest that the tested compounds may inhibit fungal pathogenicity by interfering with critical protein functions required for infection and development.

The *Pst11215* protein, a haustorium-specific effector of *P. striiformis* f. sp. tritici, was selected as a key molecular target due to its essential role in pathogen virulence^42^. This effector protein has been shown to manipulate host cellular processes by interacting with the wheat voltage-dependent anion channel (TaVDAC1), a regulator of plant defense. Through ubiquitination mediated by the E3 ligase *TaVDIP1, Pst11215* suppresses the accumulation of reactive oxygen species (ROS) in host mitochondria, thereby weakening plant immunity and enhancing pathogen infection^43^. Reactive oxygen species (ROS) are crucial in plant metabolism, acting as agents that cause cellular damage and essential signaling molecules. So, given its central role in disrupting host defense mechanisms, *Pst11215* represents a promising target for structure-based inhibitor design.

The strong binding affinities observed in this study indicate that the identified compounds may act as potential inhibitors of *Pst11215*, thereby restoring host defense responses and limiting pathogen development. These findings are consistent with previous studies^44,45^ that emphasized the importance of targeting virulence-associated proteins through molecular docking to better understand antifungal activity.

Furthermore, the identified compounds—dieckol, β-carotene, fucoxanthin, rutin, eckol, naringenin, chitinase, and viridin—can be considered promising natural sources of antifungal agents. This is supported by earlier research^46^, which demonstrated that plant-derived extracts significantly reduced disease incidence and severity compared to untreated controls. The growing interest in environmentally friendly disease management strategies highlights the importance of such natural compounds as alternatives to synthetic fungicides^26,46^.

Stripe (yellow) rust, caused by *Puccinia striiformis* f. sp. tritici (Pst), is a serious disease of wheat occurring in most wheat areas with cool and moist weather conditions during the growing season.The basidiomycete fungus is an obligate biotrophic parasite that is difficult to culture on artificial media Overall, the findings of this study confirm that SNP-based molecular identification is a reliable approach for distinguishing aggressive *P. striiformis* races. In addition, Wanquan et al.^47^ reported that the stripe (yellow) rust, caused by *Puccinia striiformis* f. sp. *tritici*, is an obligate biotrophic parasite that is difficult to culture on artificial media. The application of biological agents, including *S. latifolium*, *T. harzianum* and chitosan nanoparticles, significantly reduced disease severity and improved wheat productivity. The integration of molecular identification, biocontrol strategies, and computational docking provides a comprehensive and sustainable framework for managing wheat stripe rust in an eco-friendly manner.

## Conclusion

Wheat production worldwide continues to face significant economic losses due to rust diseases, particularly stripe rust caused by *P. striiformis*. Among available management strategies, host-plant resistance remains the most effective, economical, and environmentally sustainable approach, especially when combining race-specific and non-race-specific resistance mechanisms. However, the continuous evolution of the pathogen necessitates ongoing monitoring and the identification of new virulent races. In the present study, five aggressive *P. striiformis* isolates—111E255, 175E255, 191E255, 239E255, and 246E175—were successfully identified using molecular approaches and deposited in *GenBank* under accession numbers PV983376–PV983380. These isolates exhibited high virulence and were able to overcome several resistance genes at the adult plant stage, highlighting the importance of considering host–pathogen–environment interactions in disease management strategies.The evaluation of biological control agents demonstrated that *S. latifolium*, *T. harzianum*, and chitosan nanoparticles effectively reduced stripe rust severity under field conditions. Among these, *S. latifolium* showed particularly strong antifungal activity, likely due to its bioactive compounds. The molecular docking analysis further supported these findings, revealing that compounds such as dieckol, β-carotene, fucoxanthin, rutin, eckol, naringenin, chitinase, and viridin can bind specifically to key pathogenic proteins in *P. striiformis*, potentially inhibiting its virulence mechanisms.Both preventive and curative applications of these biological agents proved effective in reducing disease development, indicating their potential as sustainable alternatives to chemical fungicides. Moreover, these natural compounds represent promising sources of antifungal agents and may serve as lead candidates for the development of novel, eco-friendly agrochemicals. Overall, this study highlights the importance of integrating molecular identification, host resistance, and biological control strategies for effective and sustainable management of wheat stripe rust. Future research should focus on large-scale field validation, formulation development, and the exploration of additional bioactive compounds to enhance disease control efficiency.

## Supplementary Information

Below is the link to the electronic supplementary material.

Supplementary Material 1

Supplementary Material 2

## Data Availability

The raw data will be available on request. Correspondence and requests for materials should be addressed to H.S.O. The nucleotide sequence of the five aggressive races have been deposited in *GenBank* database under the accession numbers PV983376, PV983377, PV983378, PV983379 and PV983380 will release through few days and this sequence will be available on request. Correspondence and requests for materials should be addressed to H.S.O.
