# Supplementary material for: Identification of physiological races of Puccinia striiformis f. sp. tritici and molecular docking of some biological treatments as prospective fungal inhibitor candidates in wheat
Source: Sci Rep. 2026 May 6;16:14423. doi: 10.1038/s41598-026-50602-2 (PMC13149999; doi:10.1038/s41598-026-50602-2)
Supplement: Supplementary file 1 — Supplementary Material 1 [file 41598_2026_50602_MOESM1_ESM.docx]

# Table 1. Targeted ligands of biological agents for molecular docking through online databases and scientific literature.

| **Source** | **Compound** | **Link** | **Reference** |
| --- | --- | --- | --- |
| *Sargassum latifolium* | Fucoidan / Alginate / Laminarin | <https://doi.org/10.1007/s10811-021-02453-9> | Fawzy, M. A., Gomaa, M., & Hifney, A. (2021). Extraction and characterization of alginate and fucoidan from Sargassum latifolium using citric acid. Journal of Applied Phycology, 33, 2801–2810. |
| *Sargassum latifolium* | Fucoxanthin / Beta-Carotene | <https://doi.org/10.3390/md18070344> | Zhang, B., Ruan, Z. W., Luo, D., Zhu, Y., Ding, T., Sui, Q., & Lei, X. (2020). Unexpected enhancement of HDACs inhibition by MeS substitution at C-2 position of fluoro largazole. Marine Drugs, 18(7), 344. |
| *Sargassum latifolium* | Sargaquinoic acid / Sargachromenol | <https://doi.org/10.1016/j.bmcl.2004.07.062> | Lee, E. J., Lee, H. J., Park, H. J., Min, H. Y., Suh, M. E., Chung, H. J., & Lee, S. K. (2004). Induction of G2/M cell cycle arrest and apoptosis by a benz [f] indole-4, 9-dione analog in cultured human lung (A549) cancer cells. Bioorganic & medicinal chemistry letters, 14(20), 5175-5178. |
| *Sargassum latifolium* | Rutin / Gallic Acid / Caffeic Acid | <https://www.mdpi.com/1660-3397/14/7/138> | Ruocco, N., Costantini, S., & Costantini, M. (2016). Blue-print autophagy: potential for cancer treatment. Marine drugs, 14(7), 138. |
| *Sargassum latifolium* | Palmitic Acid / Stearic Acid | <https://doi.org/10.3390/md18070344> | Zhang, B., Ruan, Z. W., Luo, D., Zhu, Y., Ding, T., Sui, Q., & Lei, X. (2020). Unexpected enhancement of HDACs inhibition by MeS substitution at C-2 position of fluoro largazole. Marine Drugs, 18(7), 344. |
| *Sargassum latifolium* | Fucoxanthin | <https://doi.org/10.1016/j.nutres.2011.05.002> | Hahm, S. W., Park, J., & Son, Y. S. (2011). Opuntia humifusa stems lower blood glucose and cholesterol levels in streptozotocin-induced diabetic rats. Nutrition Research, 31(6), 479-487. |
| *Sargassum latifolium* | Eckol | <https://doi.org/10.1016/j.freeradbiomed.2004.08.012> | James, R. W., & Deakin, S. P. (2004). The importance of high-density lipoproteins for paraoxonase-1 secretion, stability, and activity. Free Radical Biology and Medicine, 37(12), 1986-1994. |
| *Sargassum latifolium* | Rutin | <https://doi.org/10.1016/j.foodchem.2017.02.119> | Lin, Y. R., Huang, M. F., Wu, Y. Y., Liu, M. C., Huang, J. H., Chen, Z., ... & Liang, S. S. (2017). Reductive amination derivatization for the quantification of garlic components by isotope dilution analysis. Food Chemistry, 230, 1-5. |
| *Sargassum latifolium* | Laminaran / Laminarin | <https://doi.org/10.1016/j.carbpol.2010.11.028> | Dotto, G. L., & Pinto, L. D. A. (2011). Adsorption of food dyes onto chitosan: Optimization process and kinetic. Carbohydrate Polymers, 84(1), 231-238. |
| *Sargassum latifolium* | Sargachromenol | <https://doi.org/10.1248/bpb.30.708> | Kim, J. H., Ha, H. C., Lee, M. S., Kang, J. I., Kim, H. S., Lee, S. Y., ... & Shim, I. (2007). Effect of Tremella fuciformis on the neurite outgrowth of PC12h cells and the improvement of memory in rats. Biological and Pharmaceutical Bulletin, 30(4), 708-714. |
| *Sargassum latifolium* | Fucoidan | <https://doi.org/10.1016/j.carbpol.2009.05.032> | Amarasekara, A. S., & Owereh, O. S. (2009). Homogeneous phase synthesis of cellulose carbamate silica hybrid materials using 1-n-butyl-3-methylimidazolium chloride ionic liquid medium. Carbohydrate Polymers, 78(3), 635-638. |
| *Sargassum latifolium* | Beta-Carotene | <https://doi.org/10.1016/j.algal.2015.07.003> | Murphy, T. E., Kapili, B. J., Detweiler, A. M., Bebout, B. M., & Prufert-Bebout, L. E. (2015). Vertical distribution of algal productivity in open pond raceways. Algal Research, 11, 334-342. |
| *Sargassum latifolium* | Ellagic acid | <https://doi.org/10.1016/j.foodres.2015.05.012> | Zivkovic, M., Hidalgo-Cantabrana, C., Kojic, M., Gueimonde, M., Golic, N., & Ruas-Madiedo, P. (2015). Capability of exopolysaccharide-producing Lactobacillus paraplantarum BGCG11 and its non-producing isogenic strain NB1, to counteract the effect of enteropathogens upon the epithelial cell line HT29-MTX. Food Research International, 74, 199-207. |
| *Sargassum latifolium* | Alginate | <https://doi.org/10.1016/j.carbpol.2009.07.002> | Putseys, J. A., Derde, L. J., Lamberts, L., Goesaert, H., & Delcour, J. A. (2009). Production of tailor made short chain amylose–lipid complexes using varying reaction conditions. Carbohydrate Polymers, 78(4), 854-861. |
| *Sargassum latifolium* | D-glucosamine | <https://doi.org/10.1016/j.jff.2012.06.001> | Luthria, D. L. (2012). Optimization of extraction of phenolic acids from a vegetable waste product using a pressurized liquid extractor. Journal of functional foods, 4(4), 842-850. |
| *Sargassum latifolium* | Suberic acid / Margaric acid / Stearic acid / Caproic acid / Palmitic Acid | <https://doi.org/10.1016/j.foodchem.2009.04.011> | Xie, J., Sun, B., Wang, S., & Ito, Y. (2009). Isolation and purification of nootkatone from the essential oil of fruits of Alpinia oxyphylla Miquel by high-speed counter-current chromatography. Food chemistry, 117(2), 375-380. |
| *Trichoderma harzianum* | Chitinase | <https://doi.org/10.1016/j.biocontrol.2006.06.005> | Krauss, U., ten Hoopen, G. M., Hidalgo, E., Martínez, A., Stirrup, T., Arroyo, C., ... & Palacios, M. (2006). The effect of cane molasses amendment on biocontrol of frosty pod rot (Moniliophthora roreri) and black pod (Phytophthora spp.) of cocoa (Theobroma cacao) in Panama. Biological control, 39(2), 232-239. |
| *Trichoderma harzianum* | Chitosan / Chitooligosaccharides / Chitohexaose / Chitotetraose | <https://doi.org/10.1016/j.ijbiomac.2016.02.031> | Zhuang, C., Xu, N. W., Gao, G. M., Ni, S., Miao, K. S., Li, C. K., ... & Xie, H. G. (2016). Polysaccharide from Angelica sinensis protects chondrocytes from H2O2-induced apoptosis through its antioxidant effects in vitro. International journal of biological macromolecules, 87, 322-328. |
| *Trichoderma harzianum* | Gliotoxin | <https://doi.org/10.1016/j.micres.2007.01.004> | Spadaro, D., Sabetta, W., Acquadro, A., Portis, E., Garibaldi, A., & Gullino, M. L. (2008). Use of AFLP for differentiation of Metschnikowia pulcherrima strains for postharvest disease biological control. Microbiological Research, 163(5), 523-530. |
| *Trichoderma harzianum* | Trichokonin VI | <https://doi.org/10.1016/j.peptides.2009.07.018> | Leite, L. H., Lacerda, A. C. R., Balthazar, C. H., Marubayashi, U., & Coimbra, C. C. (2009). Central angiotensin AT1 receptors are involved in metabolic adjustments in response to graded exercise in rats. Peptides, 30(10), 1931-1935. |
| *Trichoderma harzianum* | 6-PENTYL_ALPHA_PYRONE | <https://doi.org/10.1016/j.jbiotec.2004.02.012> | Liu, Y., Yang, S. F., Li, Y., Xu, H., Qin, L., & Tay, J. H. (2004). The influence of cell and substratum surface hydrophobicities on microbial attachment. Journal of biotechnology, 110(3), 251-256. |
| *Trichoderma harzianum* | Viridin | <https://doi.org/10.1016/j.tet.2005.09.032> | Khan, M. W., & Reza, A. M. (2005). Palladium mediated synthesis of isoindolinones and isoquinolinones. Tetrahedron, 61(47), 11204-11210. |
