# Supplementary figures and images for: Identification of physiological races of Puccinia striiformis f. sp. tritici and molecular docking of some biological treatments as prospective fungal inhibitor candidates in wheat
Source: Sci Rep. 2026 May 6;16:14423. doi: 10.1038/s41598-026-50602-2 (PMC13149999; doi:10.1038/s41598-026-50602-2)

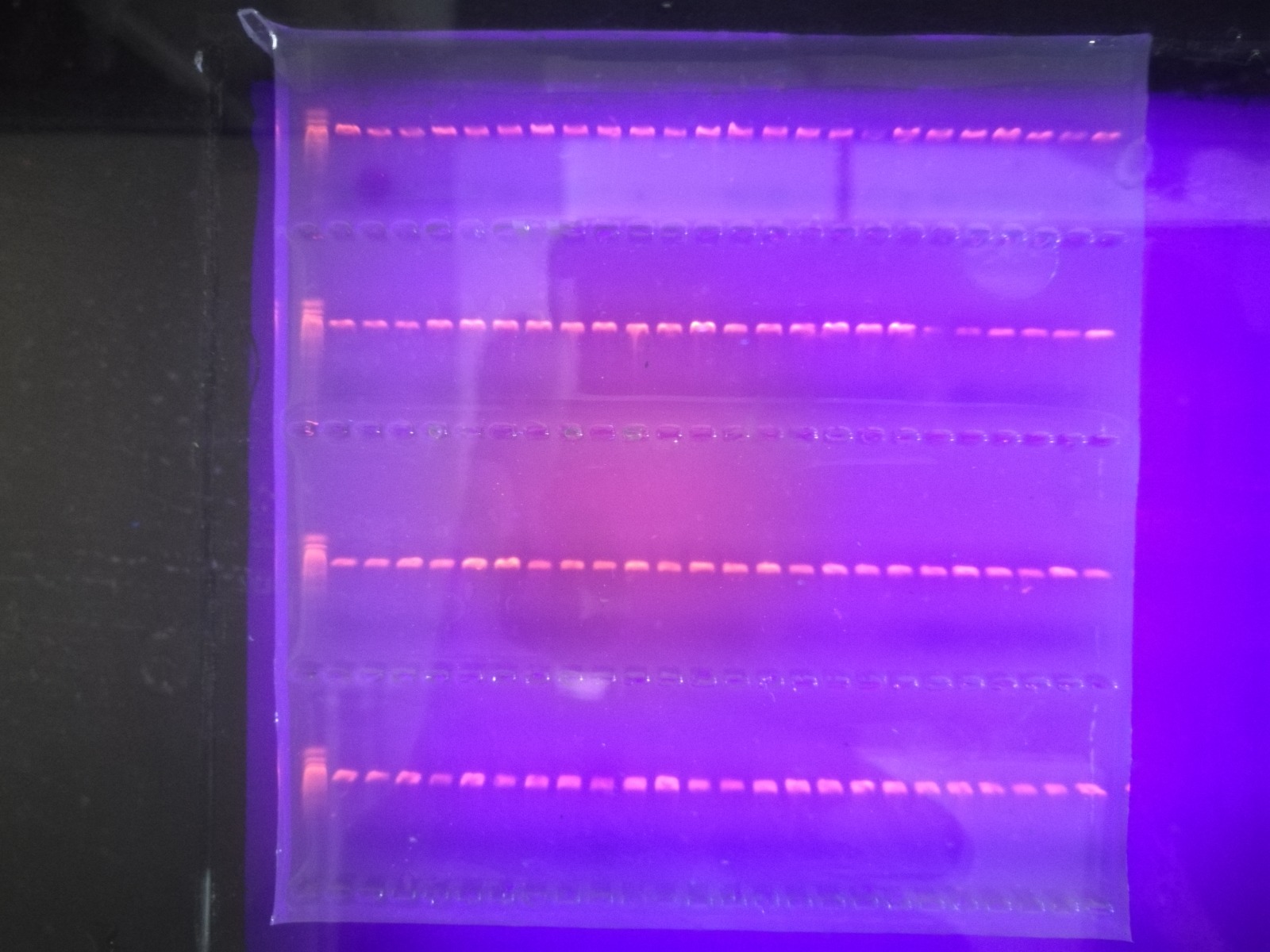


**M 1 2 3 4 5 1 2 3 4 5 1 2 3 4 5 1 2 3 4 5 1 2 3 4 5**

**100**

**300**

**400**

**2 3 4 5**

Supplement: Supplementary file 2 — Supplementary Material 2 [file 41598_2026_50602_MOESM2_ESM.docx]
